# Sugar lowering in fermented apple-pear juice orchestrates a promising metabolic answer in the gut microbiome and intestinal integrity

**DOI:** 10.1016/j.crfs.2024.100833

**Published:** 2024-09-05

**Authors:** Ali Zein Alabiden Tlais, Andrea Polo, Lena Granehäll, Pasquale Filannino, Olimpia Vincentini, Francesca De Battistis, Raffaella Di Cagno, Marco Gobbetti

**Affiliations:** a Faculty of Agricultural, Environmental and Food Sciences, Free University of Bolzano-Bozen, 39100, Bolzano, Italy; b International Center on Food Fermentation, 39100, Bolzano, Italy; c Department of Soil, Plant and Food Science, University of Bari Aldo Moro, 70121, Bari, Italy; d U.O Alimentazione, Nutrizione e Salute, Dipartimento Sicurezza Alimentare, Nutrizione e Sanità Pubblica Veterinaria, Istituto Superiore di Sanità, 00161, Roma, Italy

**Keywords:** Lactic acid bacteria, Yeasts, Sugar reduction, Mannitol, SHIME

## Abstract

Excessive sugar consumption in young people, who are the major consumers of sugary drinks, combined with limited physical activity, is an important determinant of obesity. Despite their natural appeal, fruit juices have a similar sugar content to that of sugary drinks and once metabolized, they may induce the same biological response. This study aimed to verify whether fermentation processes can make juice consumption healthier and whether reduced-sugar juices have a specific impact on intestinal function. We designed a tailored fermentation of apple-pear juices with lactic acid bacteria and yeasts, which resulted in a reduction of sugar content (27–66%) and caloric intake, and an increase in mannitol content. The impact of newly developed apple-pear juices on gut microbiome composition and functionality was evaluated *in vitro* using the Simulator of the Human Intestinal Microbial Ecosystem (SHIME). Promising changes were found in the gut microbiota and its metabolic responses and functionality, targeting pathways related to obesity and weight loss (lipopolysaccharide and secondary metabolite biosynthesis, polycyclic aromatic hydrocarbon degradation, and amino sugar and nucleotide sugar metabolism). Additionally, the fermented apple-pear juices positively modulated the intestinal epithelial features. While the simulation of the study simplifies the complex *in vivo* conditions, it suggests that low-sugar fermented apple-pear juices can elicit targeted responses in the gut ecosystem, contributing to healthier alternatives to traditional fruit juices.

## Highlights

• Three tailored fermentations of fruit juices with selected lactic acid bacteria and yeasts were investigated.
• Fermented juices resulted in a reduction of sugar content and caloric intake, and an increase in mannitol content.
• Feeding the SHIME with the fermented juices modifies SCFA profiles.
• Feeding the SHIME with fermented juices reshapes colon microbiome.
• Low sugar fermented juices exert a protective effect towards oxidative stress at intestinal level.

## Graphical abstract

**Figure:**
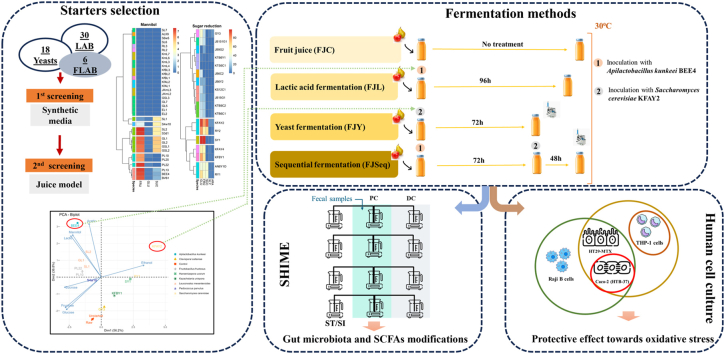

## 1 Introduction

The excessive sugar consumption has been linked to a rise in the prevalence of non-communicable diseases (NCDs) with consequent concern for public health worldwide (Bellisle et al., 2018; Pepin et al., 2019). Children and adolescents are the largest consumers of sugary drinks which, together with sweets, fast or junky food and limited physical movement, constitute the determinants of obesity (Kerkadi et al., 2019). Overweight and obesity are increasing at a rapid rate, with estimates of 60% of adults and nearly one in three children in the European Region are affected. Given these implications, the World Health Organization (WHO) published a guideline recommending the daily intake of “free sugars” from foods and drinks of no more than 10% of the total energy intake, with a further reduction to below 5% or roughly 25 g (6 teaspoons) per day would provide additional health benefits. Nevertheless, the global sugar consumption exceeds such levels (World Health Organization, 2015, 2022). More recently EFSA provided a scientific advice on a tolerable upper intake level (UL) or a safe level of intake for dietary (total/added/free) sugars based on available data on chronic metabolic diseases, pregnancy-related endpoints and dental caries (Turck et al., 2022). The document states that the intake of added and free sugars should be as low as possible in the context of a nutritionally adequate diet.

Free sugars include sugars naturally present in food, as well as all monosaccharides and disaccharides added by the producer or consumer, collectively providing caloric inputs. Although natural, the fruit juices have a sugar content similar to that of sugary drinks, and once metabolized, they can induce the same biological response (Guasch-Ferré and Hu, 2019). However, natural fruit juices are increasingly becoming valid substitutes for fresh fruits due to longer shelf-life and convenience of use raising a risk for an unconscious excessive intake of free sugars and calories when consumed. Therefore, the question arises how to make the natural fruit juices consumption heathier. Variability in fruit juice types, doses, study durations, and measured effects creates challenges in providing conclusive evidence (Pepin et al., 2019). Moderate natural juice consumption is associated with decreased cognitive decline and reduced cardiovascular risks due to vital nutrients (Bellisle et al., 2018). Conversely, high-calorie diets are linked to gut microbiome imbalance, promoting an obesity-associated microbial structure, and disrupting the intestinal barrier, leading to inflammation and various diseases (Ribeiro et al., 2017; Yuan et al., 2019; Faruque et al., 2019).

This scenario supports the importance of strategies aimed at reducing the surplus of sugar content in beverages and juices. Indeed, numerous countries around the world have imposed taxes on sugary drinks (Julia et al., 2015; Barrientos-Gutierrez et al., 2017). This political input further addresses the industrial world to develop new fruit-based drinks with a reduced sugar content while retaining the nutritional and sensory properties of the fresh fruit. Nowadays, available technological processes such as dilution with water, membrane filtration, and enzymatic digestion are far from being effective, natural and sustainable. These techniques are either expensive and high energy consuming or may affect the availability and concentration of healthy bioactive compounds and aroma and flavors molecules (Cywińska-Antonik et al., 2023). On the other hand, the addition of artificial sweeteners (e.g., aspartame and sucralose) to improve the sensory taste affected by these techniques has been additionally employed, but it is limited by several regulations since their effects on human health are still debatable. Facing this background, polyalcohols including mannitol, erythritol and xylitol emerged as healthier alternatives. They are sugar derivatives and are classified as natural sweeteners which do not contain significant calories and have not known health implications (Chattopadhyay et al., 2014; Mooradian et al., 2017). Since fermentation of apple and pear juices, among its multiple effects (i.e. enhancing nutritional, antioxidant, and beneficial health properties) (Li et al., 2021; Wang et al., 2021; Zhang et al., 2021), reduces sugars and promotes the synthesis of poly-alcohols, it has been recognized as a suitable option to meet the growing demand for natural and healthy fruit juice formulations, while also being a cost-effective and sustainable technology.

We hypothesized that the metabolic potential of lactic acid bacteria (LAB) and yeasts can be driven to naturally reduce the sugar content of apple-pear juices during the fermentation. Plenty data from human studies and animal model suggests that obesity and related diseases are influenced by gut microbiota-derived metabolites and are often associated with profound gut dysbiosis (Geng et al., 2022). Thus, after the definition of the fermentation protocol for reducing the sugar content of fruit juices, we explored their impact on the structure, behavior and functionality of gut microbiota through the simulator of the human intestinal microbial ecosystem (SHIME) that mimics representative gut ecosystems and the entire gastrointestinal transit. Additionally, stipulated that healthy intestinal epithelial cells play important role in mitigating intestinal disorders (Ghosh et al., 2021), the protection effect of low-sugar fermented apple-pear juices was evaluated *in vitro* using several human intestinal cell lines used as mono- and tri-culture models as well the capability to adsorb glucose. Exploring and describing the cause-effect relationship between the intake of new apple-pear juices with lower sugar content, gut ecosystem evolution and intestinal epithelial features, we believe our findings can contribute to supply healthier alternatives to traditional juices.

## 2 Materials and methods

### 2.1 Plant materials, microorganisms, and culture conditions

Pasteurized apple and pear juices supplied by Zuegg Com (Lana, Bolzano, Italy) and Zipperle (Merano, Bolzano, Italy) were stored at 4 °C prior the use. Thirty-six strains of LAB and eighteen of yeasts from the Culture Collection of the Department of Soil, Plant and Food Science, University of Bari Aldo Moro (Bari, Italy) and the Culture Collection of Micro4Food laboratory of Faculty of Agricultural, Environmental and Food Sciences, Libera Università di Bolzano, Bolzano, Italy were used in this study (Table S1). Cultures of LAB and fructophilic lactic acid bacteria (FLAB) were stored as stocks in 20% (v v ^−1^) glycerol at – 20 °C and regularly propagated in MRS broth (Oxoid, Basingstoke, Hampshire, United Kingdom) and FYP broth at 30 °C for 24 h, respectively. Yeasts were propagated at 25 °C for 24 h in SAB broth (Oxoid).

### 2.2 Starter screening for carbohydrate metabolism

Aiming to select starters with high metabolic potential suitable for different fruit juices, five synthetic media differed in the main carbon source (glucose, fructose, sucrose, xylose, and arabinose) were used as substrates to investigate the microbial metabolism of carbohydrates and polyalcohols, and the production of organic acids and ethanol. Synthetic media were obtained by mixing 10 g L^−1^ of any of former carbon sources with 5 g L^−1^ bacteriological peptone, 10 g L^−1^ yeast extract, 0.5 g L^−1^ TweenTM (Oxoid Ltd, Basingstoke, Hampshire, England), 2 g L^−1^ sodium acetate trihydrate, 0.2 g L^−1^ MgSO_4_, 0.01 g L^−1^ FeSO_4_, 0.01 g L^−1^ MnSO_4_, and 0.01 g L^−1^ NaCl. Starters were inoculated in synthetic media to a final cell density corresponding to ca. 7.0 Log CFU mL^−1^ for LAB and FLAB, and ca. 5.0 Log CFU mL^−1^ for yeasts. After inoculum, all media were incubated at 30 °C for 24 h. The optical density was used to track microbial growth throughout the incubation. The wavelengths used were 620 nm and 600 nm for bacteria and yeasts, respectively. Data were modelled using four different equations, namely, Richards, Gompertz, Logistic and Grompertz exponential (Zwietering et al., 1990). The main parameters taken into consideration to assess the kinetics of microbial growth are the following: A, which represents the difference between the OD at the time of inoculation and that in the stationary phase; μ_max_ (OD unit h^−1^), which is the maximum growth rate; λ (h), which is the length of the lag phase. Growth capacity was also determined by integrating the area under the curve (AUC). In order to determine the consumption of carbohydrates and the synthesis of lactic acid, ethanol, and polyalcohols, samples derived from different synthetic media after 24 h incubation were centrifuged (10,000 rpm for 10 min) and then filtered through a 0.22 μm filter (Millipore Corporation, Bedford, MA, USA). Concentrations of glucose, fructose, sucrose, xylose, arabinose were determined through high performance liquid chromatography (HPLC) analysis equipped with a Spherisorb column (Waters, Millford, USA) and a PerkinElmer 200a refractive index detector. Mannitol, erythritol, xylitol, and acetic, lactic and citric acids, and ethanol were determined by HPLC equipped with an Aminex HPX-87H column (ion exclusion, Biorad Los Angeles, California), a PerkinElmer 200a refractive index detector, and UV detector operating at 210 nm (Tlais et al., 2022). Carbohydrates, organic acids and ethanol standards were purchased from Sigma-Aldrich (Steinheim, Germany). Strains showing better metabolic performances were used for further investigation on fruit juice. Due to low pH of apple juice (3.64 ± 0.05), blending with pear juice (pH of 4.56 ± 0.08) was required. Subsequently, the formulated apple (30%) - pear (70%) juice, was singularly inoculated with ca. 7.0 Log CFU mL^−1^ of LAB and FLAB, and ca. 5.0 Log CFU mL^−1^ of yeasts. The fermentation was carried out at 30 °C for 72 h. The juice without bacterial inoculums was cultured under the same conditions and used as the control. Samples were taken before and after fermentation. Carbohydrates reduction, and release of organic acids, polyalcohols and ethanol were evaluated as previously described.

### 2.3 Laboratory-scale juice fermentation and characterization

Fermentation of apple-pear juice was carried out in laboratory scale. *Apilactobacillus kunkeei* BEE4 and *Saccharomyces cerevisiae* KFAY2, selected as starters, were added at an initial cell density of ca. 7 Log CFU mL^−1^ (FLAB) and ca. 5 Log CFU mL^−1^ (yeast) following different fermentation methods (Fig. S1). One method included the lactic fermentation at 30 °C for 96 h (FJL) whereas another included only yeast fermentation at 30 °C for 72 h (FJY). The sequential fermentation (FJSeq) included the inoculum of FLAB and yeast strains temporally deferred. *A. kunkeei* BEE4 was inoculated at the beginning of the fermentation, and *S. cerevisiae* KFAY2 after 3 days. Apple-pear juice, without microbial inoculum and incubation, was used as the control (FJC). As expected, when *S. cerevisiae* was inoculated, ethanol production occurred. The removal of ethanol was achieved using an optimized evaporation technique (50 °C). To compensate for volume reduction and changes in sugar content, water was added to restore original volume and sugar concentrations without ethanol. All samples after fermentation were filtered and pasteurized (90 °C for 10 min). Carbohydrates reduction, and production of organic acids, mannitol and ethanol were determined as described in previous section. Energy value was determined by the accredited Chelab S.r.l (Merieux NutriSciences Corporation, Resana TV, Italy).

### 2.4 SHIME experiment

A 4-SHIME (ProDigest, Ghent, Belgium) configuration was built. It consists in four parallel SHIME units, each one including three consecutive bioreactors that simulate stomach/small intestine, proximal (PC) and distal (DC) colon, respectively. They were maintained at 37 °C and under anaerobic conditions by flushing with sterile N_2_ (Liu et al., 2021). PC and DC bioreactors were filled with 500 and 800 mL of adult L-SHIME® growth medium (Prodigest, Belgium) and internal pH was maintained in the ranges 5.6–5.9 and 6.6–6.9, respectively, through computer-controlled addition of 0.5 M HCl and 1.0 M NaOH to mimic the colon physiological conditions. In the stomach/small intestine bioreactors the pH was 2. During the simulation, all bioreactors were continuously stirred and monitored for constant volume and pH stability (Polo et al., 2022). Prior to initiation, both PC and DC bioreactors were inoculated simultaneously using the fecal material of a healthy 30-year-old volunteer. The fecal slurry was prepared as reported by (Molly et al., 1993) and inoculated (5% v/v) within 1 h after the collection. Thereafter, a static incubation of 12 h was performed to allow the initial microbiota adaptation. Then, a stabilization period (needed to evolve stable and representative colonic microbiota) and a 2-weeks steady state period (representing the experiment baseline) followed, in which each colon bioreactor was fed three times per day (every 8 h) with 200 mL of predigested adult L-SHIME® growth medium (ProDigest) at pH 2. Pre-digestion consisted of a 75 min incubation in the stomach/small intestine bioreactors with the addition of 60 mL of pancreatic juice (12.5 g NaHCO_3_, 6.0 g dehydrated bile extract and 0.9 g pancreatin per liter). During stabilization and steady state periods, 2 mL lumen samples were collected three times per week at regular times from each colon bioreactor and analyzed for short chain fatty acid (SCFA) concentrations to monitor the fermentative activity of the microbial communities, and to verify the stability and reproducibility of the microbial ecosystems. Then, a 2-weeks treatment period started in which the feeding of colon bioreactors was supplemented with 200 mL/day (i.e. 66.7 mL, 3 times a day) of juice (a different juice for each SHIME line). Before (T0) and during feeding with juices, lumen (40 mL) samples were collected three times a week at regular times from each colon bioreactor, for all SHIME units. Lumen samples were stored at −80 °C until the analysis. The donor of fecal material for the inoculum of colon bioreactors was selected from a cohort of typical Mediterranean diet (MD) consumers as described by Da Ros et al. (2021). To this purpose, an initial cohort of 40 healthy volunteers aged between 19 and 50 years, no evidence of pathologies, no history of drug use in the last 6 months, no smokers, no regular alcohol consumers, not pregnant, and who had been antibiotic and probiotic free in the 6 months prior to the sample collection was recruited with fully informed written consent from all volunteers. The volunteers had a Mediterranean diet score (MDS, which ranges from 0 to 8 points for minimal to maximal adherence, respectively) from 4 to 8, calculated through the administration of a nutritional questionnaires about dietary habits and frequencies of food consumption (Wareham et al., 2003) according to Gutiérrez-Díaz et al. (2016). Fecal material was collected from all volunteers into sterile bags and added (ratio 1:2) with RNA later solution (Applied Biosystems, Foster City, CA, USA) at 4 °C. The mixture was immediately homogenized into sterile bags using a stomacher apparatus (Stomacher 400 Circulator, Seward, United Kingdom) and analyzed for their microbiota composition and SCFAs content. The selection of the donor was based on the clustering of fecal microbiota abundances of the volunteers, aggregated at family level, together with SCFA data. Clustering was performed with the Manhattan distance matrix and Ward D2 method as described by Da Ros et al. (2021). Partial least-squares discriminant analysis (PLSDA) was performed considering the adherence to MD as an independent variable and OUT abundances as feature for the model. The contribution of each feature was further explored and annotated with the explanatory independent variable level. All statistical analyses were performed in R programming version 4.04 (R Foundation, Vienna, Austria). The collection of data from consumers and the use of human fecal samples were approved by the Ethics Committee of the Free University of Bozen on July 17, 2019, and informed consent for the experimentation was obtained from all subjects involved in the study.

### 2.5 DNA extractions, metataxonomic and metagenomic analyses

DNA was extracted, in triplicate, from lumen samples (6 mL aliquots) collected from different colon compartments (both PC and DC) during the 4-SHIME experiment by using FastDNA Spin Kit For Soil (MP Biomedicals, Italy), according to the manufacturer instructions. The assessment of the DNA concentration was with the fluorimeter Qubit 2.0 (Invitrogen, Italy). DNA processing was performed at the Genomics Platform–Unit of Computational Biology (Edmund Mach Foundation, TR, Italy), DNA purification, library preparation, and sequencing Illumina MiSeq platform, paired-end strategy, following standard protocols. Paired-end reads were preprocessed using fastp (Chen et al., 2018), with adapter removal and a quality filter of 20. After quality filtering, samples below 10,000,000 read pairs were removed. Species-level taxonomic classification was done with Metaphlan4 (Blanco-Míguez et al., 2023) with the mpa_vJan21_CHOCOPhlAnSGB_202103 database, including the “-t rel_ab_w_read_stats” option to list absolute abundance for alpha diversity.

### 2.6 Short chain fatty acids (SCFA) quantification

Metabolic profile of SCFA (acetate, propionate and butyrate) was determined on lumen samples by HPLC equipped with an Aminex HPX-87H column, a PerkinElmer 200a refractive index detector and UV detector operating at 210 nm using the same instrumental method previously described (Bondue et al., 2020).

### 2.7 Advanced human cell culture models and functional evaluations

The human colon adenocarcinoma Caco-2 (HTB-37) and THP-1 cell lines were obtained from American Type Culture Collection (ATCC, Manassas, USA). Mucus-producing HT29-MTX and Human Burkitt's lymphoma Raji B cell lines were obtained from the European Collection of Authenticate Cell cultures (ECAC, UK). Caco-2, HT29-MTX, and Raji B cells were cultured as described by Vincentini et al. (2022). THP-1 cells were cultured and maintained according to Bisht et al. (2020). Caco-2/HT29-MTX/Raji B cells triculture were obtained by seeding Caco-2/Ht-29 MTX cells (9:1 ratio) co-culture at a density of 2.25 × 10^5^ cells/cm^2^ on Polyethylene Terephthalate Transwells® insert (PET) with a pore size of 1 μm (Cellquart), allocated in 12 well culture plate. The apical (Ap) and the basolateral (Bl) compartments were respectively filled with 0.5 and 1.5 mL complete culture medium. Culture medium was changed every two days. At the 14th, 5 × 10^5^ Raji B cells were added, in the Bl side of each insert in DMEM:RPMI (1:1) culture medium and removed and readded at 16th and 19th day of co-culture, to allow M phenotype induction in Caco-2 cells. THP-1 cells (4 × 10^5^) were seeded in 2 mL of RPMI in 12-well plates and differentiated with 20 ng mL^−1^ PMA (phorbol-12-myristate-13-acetate; P1585, Sigma) for 48 h. The PMA was then removed and washed with PBS and the cells were rested for 24 h in RPMI medium. Cell differentiation is verified by evaluating cell adhesion and spreading under an optical microscope.

For the inflammatory triculture model, Human Caco-2 and HT29-MTX cells (2 × 10^4^ in total) were seeded at a 9:1 ratio in 6.5-mm Transwell® with 1-μm pore polyester membrane inserts (Cellquart) and allowed to grow and differentiate for 20 days from seeding. The culture medium (0.5 μL in the apical and 1.5 mL in the basolateral compartments) was replaced every 2 days. On the 20th day of culture, epithelial cells were moved into the well containing differentiated macrophagic THP-1 cells, then the basolateral medium was replaced with 400 μL of RPMI. After assembly the triculture was rested for 24 h in preparation for pro-inflammatory induction. Cell treatments were performed by freeze drying and resuspending the juices (10 mL) in DMEM complete medium (10 mg mL^−1^, stock solution). Samples were sterilized through 0.22 μm filter membrane (Millipore) to remove lactic acid/yeast cells and diluted in the experiments at 100 mg mL^−1^. Barrier impairment was assessed on intestinal triculture model both by Trans-epithelial Electrical Resistance (TEER) measurement, and Lucifer Yellow (LY) translocation. TEER was evaluated by a chop-stick electrode device (Millicell ERSVoltameter-Millipore-Sigma, San Luis, Mo, USA). Paracellular permeability was assessed by Lucifer Yellow CH dilithium salt (LY) 457 D (Sigma-Aldrich) translocation (Vincentini et al., 2022). Secreted cytokines were measured in medium collected both from macrophagic differentiated THP-1 cells and from triculture models treated with 10 mg mL^−1^ of each sample for 24 h alone or in combination with LPS 250 (mg/mL). The triculture inflammatory model was exposed to the samples (10 mg mL^−1^) from the apical part while the basolateral compartment was stimulated with LPS (250 mg mL^−1^). Two models were used in order to investigate how intestinal cells can modulate inflammatory response to the macrophagic differentiated cells. Specific ELISA kits from Fine test (Fine Biotech Co., Ltd. Wuhan, Hubei, China) for interleukin (IL)-6, −8, and tumor necrosis factors (TNF α, and MCP-1) were used in accordance with the manufacturers' instructions. The level of reactive oxygen species (ROS) was determined by assaying the fluorescence intensity of 2,7-dichlorofluorescein diacetate (DCF-DA). Fluorescence was read at 485/520 nm with NIVO spectrofluorometer. Glucose uptake was measured using a non-radioactive, homogeneous bioluminescent 2DG Uptake-Glo™ kit (Promega) as per manufacturer's instructions.

### 2.8 Sensory analysis

Sensory evaluation of all juices was conducted using Quantitative Descriptive Analysis (QDA) (Tlais et al., 2023) by 10 non-trained panelists. Samples, which were refrigerated and randomly coded, were served in 15 mL portions at 15 °C, along with non-salted table biscuits and still water. Sensory attributes were scored on a scale from 0 (minimum) to 10 (maximum), including appearance (color intensity and absence of defects), taste parameters (sweetness, acidity, astringency), flavor and aroma intensity, and overall rating (including all rated attributes).

### 2.9 Statistical analysis

Each analysis was performed in triplicates. Data of starters screening, juices development, and human cell lines were subjected to analysis of variance by the General Linear Model (GLM) of R statistical package. Pairwise comparison of treatment means was achieved by Tukey-adjusted comparison procedure with *p*-value (P) < 0.05. Data of sugars, mannitol, organic acids, and ethanol (during juice screening) were subjected to Principal Component (PCA) analysis. Hierarchical clustering analysis, using the default method available in R, was performed on data referring to growth parameters, sugars, mannitol, organic acids, and ethanol (during starters screening). Data of SCFAs underwent to one-way ANOVA and individual comparisons were made post hoc with the Tukey Kramer test, while the comparisons between paired groups was performed through the Student's t-test. Both analyses were computed using GraphPad Prism 5, and differences were considered significant with *p*-values (P) < 0.05. Statistical analysis and plotting of microbiome were done in R version 4.2.0 (R core team, 2022) with the phyloseq (v.1.40.0) (McMurdie and Holmes, 2013) and ggplot 2 (3.3.6) (Wickham, 2016) packages. Alpha diversity on absolute abundance was calculated with Shannon diversity index. Statistical differences in alpha diversity were determined with a Kruskal-wallis rank sum test and Dunn's test with the Benjamini–Hochberg (BH) method to adjust for multiple testing. To explore beta diversity, a principal coordinate analysis (PCoA) was calculated on species-level relative abundance, where taxa lower than 0.02% had been removed. Permutational multivariate analysis of variance (PERMANOVA) using adonis 2 from R vegan package (Oksanen et al., 2013) was used on the ordination values to determine differences in beta diversity between the groups, and a Tukey test was used as an ad hoc test. We then used linear discriminant analysis (LDA) effect size (LEfSe) (Segata et al., 2011) to determine taxa that were differently abundant between juices and time points in the different colon tracts. Functional profiling was performed by HUMAnN3v3.6 (Beghini et al., 2021), using default options in combination with the UniProt/Uniref 201901b database. The resulting gene family abundance tables were converted to KEGG orthologies (KO) and re-normalized to copies per Million (CPM). Subsequently, KO genes were regrouped into KEGG pathways using a custom KEGG pathway definition file. CPM-normalized KO tables were stratified by bacterial taxa in HUMAnN3 and summarised by pathway to determine bacterial contributions to specific pathways. Differential abundance analysis on log-transformed functional KO genes and summarised pathways from HUMAnN3 was done with MaAsLin2 (Mallick et al., 2021), comparing all fermented juices (FJL, FJY, FJSeq) with the control (FJC), and the time points T1, T2 to T0. The features were filtered with a minimum abundance 0.0001 and a minimum prevalence of 0.10. The Benjamini–Hochberg method was used to control for False Discovery Rate (FDR). Features were considered significantly different with the adjusted p-value (q < 0.05). Differentially abundant KEGG genes and pathways were visualized with pathview (Luo and Brouwer, 2013).

## 3 Results

### 3.1 Metabolic activity in synthetic media

**Fig. 1:**
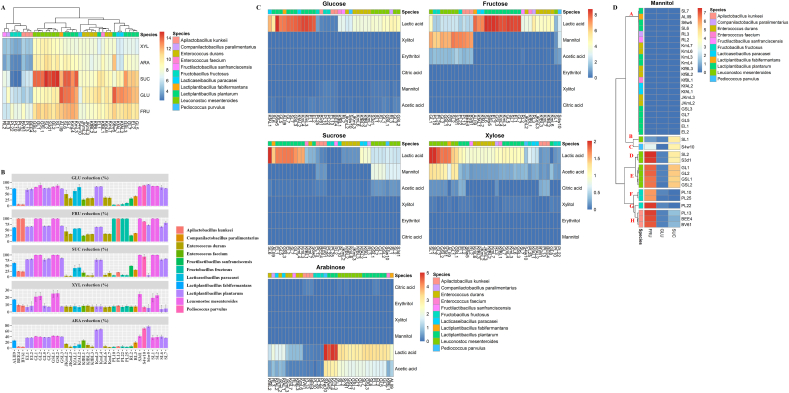
Area under the curve (OD.h) (A), sugar reduction (%) (B), lactic, acetic, and citric acids and polyalcohol (xylitol, erythritol and mannitol) production (g L^−1^) (C) in synthetic media differentiated by main carbon sources (glucose, fructose, sucrose, xylose, and arabinose) inoculated with 36 lactic acid bacteria strains after 24 h of incubation at 30 °C. Mannitol production only in glucose, fructose, and sucrose synthetic media (D).

**Fig. 2:**
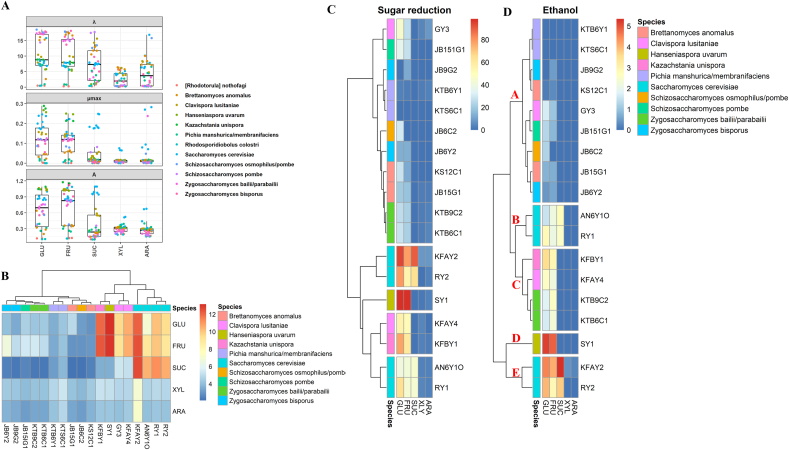
Growth parameters (maximum growth “A”, maximum growth rate “μ_max_”, lag phase “λ”) (A), area under the curve (OD.h) (B), sugar reduction (%) (C), and ethanol production (g L^−1^) (D) in synthetic media differentiated by main carbon sources (glucose, fructose, sucrose, xylose, and arabinose) inoculated with 18 yeast strains after 24 h of incubation at 30 °C.

All strains were subjected to the metabolic screening by using synthetic media. *Lactiplantibacillus plantarum*, *Lactiplantibacillus fabifermentans* and *Lacticaseibacillus paracasei* species showed the highest values of AUC in glucose based medium. Fructose as a carbon source led to less variation among strains, with *L. plantarum* SL6 showing the highest AUC. Except for *Fructobacillus fructosus*, all LAB species adapted and grew well in sucrose-based medium. Generally, lower performance was found for most strains in the growth medium containing arabinose and especially xylose (Fig. 1A). Sugar reduction almost mirrored the growth capacity. *Leuconostoc mesenteroides* strains mostly showed the highest reduction of sugars (25–100 %) among different media. As expected, *F. fructosus* and *A. kunkeii* showed distinctive preference to fructose over other carbon sources. *Pediococcus parvulus* S4w10 and *L. fabifermentans* ALII9, as well as several strains of *L. plantarum*, demonstrated considerable capacity for reducing sugars, with effectiveness varying based on the specific carbon source utilized (Fig. 1B). The most prevalent glucose microbial derivative was lactic acid, followed by mannitol and acetic acid with certain strains producing traces of xylitol or erythritol. Mannitol release, together with lactic and acetic acids, distinguished *Leuc. mesenteroides*, *F. fructosus*, *A. kunkeii*, and *P. parvulus* strains from other strains in fructose-based medium. Similarly, former strains expect *F. fructosus* strains, which did not respond well to sucrose-based medium produced same metabolites. Xylose was metabolized by most strains into lactic acid and acetic acid. Arabinose was converted by most strains, with the exception of *F. fructosus* and *A. kunkeii*, solely into only lactic acid or together with acetic acid (Fig. 1C). Since only low traces of xylitol and erythritol, as polyalcohols, were found throughout this screening, our attention was drawn to mannitol synthesis. Considering the release of mannitol in glucose, fructose, and sucrose media, strains were grouped in eight clusters (Fig. 1D). Strains from cluster 1 were excluded because they did not produce mannitol. Assuming that each cluster groups strains with similar behavior, *Leuc. mesenteroides* SL1 (cluster B), *P. parvulus* S4w10 (C), *Leuc. mesenteroides* SL2 (D), *Leuc. mesenteroides* GL1 (E), *F. fructosus* PL10 (F), *F. fructosus* PL22 (G), and *A. kunkeii* BEE4 (H) were selected as representative strains for each cluster for further screening. Similarly, yeasts exhibited distinct growth kinetic profiles when inoculated in different synthetic media (Fig. 2A). Most yeasts strains were able to grow in glucose and fructose media. Only *S. cerevisiae* and *Clavispora lusitaniae* strains properly grew in sucrose medium. In xylose and arabinose media *S. cerevisiae* KFAY2 exclusively showed high AUC values (Fig. 2B). When yeasts utilized carbon sources, acetic acid at low concentrations (0.09–0.33 g L^−1^) and ethanol (0.14–5.35 g L^−1^) were found. As expected, higher sugar reduction and, as a result, ethanol production was associated with strains showing the highest growth capacity (Fig. 2C and D). Based on these tendencies, strains were grouped into five clusters (Fig. 2C and D). Regardless of cluster plots, selecting one representative strain from each cluster resulted in the following list of strains: *C. lusitaniae* GY3 (cluster A), *S. cerevisiae* RY1 (B), *Kazachstania unispora* KFBY1 (C), *Hanseniaspora uvarum* SY1 (D), and *S. cerevisiae* KFAY2 (E) (Fig. 2C and D).

### *3.2* *In situ* metabolic response

**Fig. 3:**
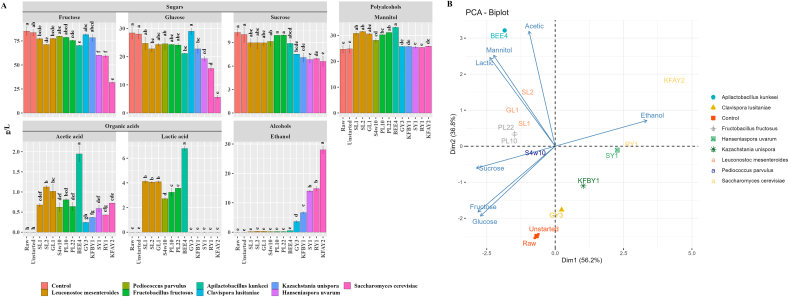
Sugars, organic acids, mannitol, and ethanol quantification (g L^−1^) in raw apple-pear juice (Raw), apple-pear juice without microbial inoculum (Unstarted), and fermented apple-pear juice, which were incubated at 30 °C for 72h (A). Principal component analysis (PCA) of sugars, organic acids, mannitol, and ethanol of raw, unstarted and fermented apple-pear juice (B). Fermentations were carried out with selected single cultures of *Leuconostoc mesenteroides* SL1, SL2, and GL1, *Pediococcus parvulus* S4w10, *Fructobacillus fructosus* PL10 and PL22, *Apilactobacillus kunkeei* BEE4, *Clavispora lusitaniae* GY3, *Kazachstania unispora* KFBY1, *Hanseniaspora uvarum* SY1, and *Saccharomyces cerevisiae* RY1 and KFAY2. Data points with different superscript letters (a–h) differ significantly (P < 0.05).

Apple-pear juice was used to investigate the metabolic performances of the selected strains. A species dependent response was found under the juice setting. When compared to the control juices, *A. kunkeei* BEE4 showed the highest significant (P < 0.05) reduction of glucose (18 and 59%) and fructose (26 and 78%) among LAB strains, and *S. cerevisiae* KFAY2 among yeasts, respectively (Fig. 3A). Only yeasts were able to significantly (P < 0.05) reduce the level of sucrose in fermented juices, whilst only LAB were able to release considerably mannitol, with *A. kunkeei* BEE4 showing the highest value. Traces amount of ethanol were found during lactic fermentation whereas higher and significant (P < 0.05) values were found when yeasts were inoculated and in particular by *S. cerevisiae* KFAY2. To visualize the distribution of raw and fermented juices based on their sugars, mannitol, organic acids, and ethanol profiles PCA was used (Fig. 3B). A general metabolic response of the similarities and differences among the strains as well as the inter-relationships among the measured parameters distinguished mainly *A. kunkeei* BEE4 for a good reduction of sugar (20%) and production of mannitol (33 g L^−1^) and *S. cerevisiae* KFAY2 due to the high reduction of sugar (65%), although an ethanol production (28 g L^−1^), as potential candidates for ongoing fermentation optimization (Fig. 3B).

### 3.3 Low-sugar fermented juice

In a laboratory-scale experiment, apple-pear juice was subjected to different biotechnological strategies. The effect of applied methods was relevant on the reduction of sugar on apple-pear juice. When compared to the FJC, selected yeast (FJY) significantly (P < 0.05) decreased fructose, glucose, and sucrose levels (Table 1). Lactic acid fermentation (FJL) caused a lower reduction in fructose, glucose, and sucrose but higher increase (P < 0.05) of mannitol than FJY. When the two selected strains were sequentially used (FJSeq) an almost intermediate sugar and mannitol levels between FJY and FJL was found (Table 1). The metabolism of carbohydrate reflected the levels of lactic and acetic acids (Table 1). The sugar content mirrored the high significant (P < 0.05) energy value seen in FJC and the lower significant values in FJY and FJSeq (Table 1).

**Table 1:** Chemical characteristics and energy value (KJ 100 g^−1^) of raw apple-pear juice (FJC), apple-pear juice fermented with *Apilactobacillus kunkeei* BEE4 for 96 h (FJL), apple-pear juice fermented with *Saccharomyces cerevisiae* KFAY2 for 72 h (FJY), and apple-pear juice fermented sequentially by *A. kunkeei* BEE4 for 72 h and then followed by *S. cerevisiae* KFAY2for 48 h (FJSeq). Fermented apple-pear juices were incubated at 30 °C. The removal of ethanol in FJY and FJSeq was achieved using an optimized evaporation technique (50 °C). Results are shown as the means (± standard deviation) of triplicate analysis. Row data with different superscript letters (a-d) differ significantly (P < 0.05).

Parameters	FJC	FJL	FJY	FJSeq
**Fructose (g L**^**−**^**^1^)**	85.2 ± 3.2^a^	66.7 ± 0.5^b^	30.5 ± 0.0^d^	43.0 ± 0.6^c^
**Glucose (g L**^**−**^**^1^)**	28.5 ± 1.5^a^	20.0 ± 0.6^b^	4.8 ± 0.1^c^	7.5 ± 0.0^c^
**Sucrose (g L**^**−**^**^1^)**	10.3 ± 0.3^a^	7.5 ± 0.1^b^	6.3 ± 0.4^c^	5.6 ± 1.3^c^
**Mannitol (g L**^**−**^**^1^)**	23.7 ± 2.0^b^	34.5 ± 0.5^a^	26.5 ± 0.2^b^	33.2 ± 0.9^a^
**Lactic acid (g L**^**−**^**^1^)**	0.00 ± 0.00^c^	10.00 ± 0.10^a^	0.00 ± 0.00^c^	8.00 ± 0.20^b^
**Acetic acid (g L**^**−**^**^1^)**	0.00 ± 0.00^c^	2.29 ± 0.00^a^	0.06 ± 0.02^c^	1.77 ± 0.12^b^
**Ethanol (g L**^**−**^**^1^)**	0.00 ± 0.00^b^	0.80 ± 0.04^a^	0.91 ± 0.04^a^	0.85 ± 0.00^a^
**Energy value (KJ 100g**^**−**^**^1^)**	230 ± 11^a^	185 ± 12^b^	133 ± 12^c^	139 ± 12^c^

### 3.4 Fecal donor

Based on the clustering analysis, a fecal donor was randomly selected as representative of the main cluster of volunteers displaying high adherence to MD (MDS >5) (Koirala et al., 2022). Gut microbiota composition of the selected donor is reported in Table S2.

### 3.5 Feeding with the fermented juices modifies SCFA profiles

The concentration of SCFA in the colon SHIME bioreactors was quantified to assess the metabolic response of the colon microbiota following the administration of various fruit juices. As SCFA before and during feeding followed a statistical trend, these and the following results only refer to the following sampling times: before feeding (T0), and at 7- and 14-days during feeding (T1 and T2, respectively) (Table 2). At T0 no statistical differences (P > 0.05) of SCFAs concentration among different SHIME units were found, both in PC and DC bioreactors. On the contrary, a significant (P < 0.05) increase in the concentration of both acetic and propionic acids was observed due to the fruit juices intake (T1 and T2) compared to before T0. In PC, the highest values of acetic acid were observed at T1, with FJC resulting in the highest level followed by FJL. At T2, the values decreased even if remained significantly higher than those before intake (T0). The only exception was the SHIME line fed with FJY that displayed the highest acetic acid concentration at T2. A similar trend was found in DC vessels, where the highest values of acetic acid were obtained after one week of FJL and FJC intake. After two weeks, a significant (P < 0.05) decrease was found only for FJL and FJC but, overall, concentrations remained still significantly (P < 0.05) higher than T0. Similarly, the propionic acid concentration significantly increased after feeding with all FJs. In this case, the highest value in PC tract was found after two weeks of feeding with FJY, while FJL intake resulted in the highest value in the in DC. No differences were found between T1 and T2, exception being PC after feeding with FJSeq and DC after feeding with FJL, which increased with longer intake (T2). Comparing different FJs at the same timepoint, no significant (P > 0.05) differences were observed nether at T1 nor at T2, with only exception of DC after one week of feeding with FJY, which showed a lower concentration. Differently, butyric acid was not found in PC tract fed with juices, with only exception after T1 of feeding with FJY. In DC tract, its concentration significantly increased after T1 of feeding, with FJSeq and FJC displaying the highest levels. However, after T2, it decreased to similar level than T0. The only exception was FJSeq, resulting in a concentration still higher than T0.

**Table 2:** Short chain fatty acids (SCFAs) concentration (mM) in proximal (PC) and distal (DC) colon reactors before (T0), after one (T1) and two (T2) weeks of treatment period. 4-SHIME® units (1, 2, 3 and 4) were treated with raw apple-pear juice (FJC), apple-pear juice fermented with *Apilactobacillus kunkeei* BEE4 for 96 h (FJL), apple-pear juice fermented with *Saccharomyces cerevisiae* KFAY2 for 72 h (FJY), and apple-pear juice fermented sequentially by *A. kunkeei* BEE4 for 72 h and then followed by *S. cerevisiae* KFAY2for 48 h (FJSeq), respectively. Fermented apple-pear juices were incubated at 30 °C.

	PC1 (FJC)	PC2 (FJL)	PC3 (FJY)	PC4 (FJSeq)	DC1 (FJC)	DC2 (FJL)	DC3 (FJY)	DC4 (FJSeq)
**Acetic acid (mM)**								
**T0**	8.08 ± 0.15 ^a/***a***^	6.98 ± 0.06^a/***a***^	8.70 ± 0.12 ^a/***a***^	6.60 ± 0.21^a/***a***^	12.51 ± 0.03^a/***a***^	12.00 ± 0.21^a/***a***^	12.62 ± 1.64^a/***a***^	11.22 ± 0.70^a/***a***^
**T1**	25.75 ± 0.30 ^d/***b***^	20.78 ± 0.27^a/***b***^	12.92 ± 0.06 ^b/***b***^	17.06 ± 1.77^c/***b***^	29.46 ± 0.67^a/***b***^	30.97 ± 0.47^a/***b***^	20.63 ± 0.05 ^b/***b***^	22.63 ± 0.21^c/***b***^
**T2**	15.61 ± 0.21^d/***c***^	12.95 ± 0.15^a/***c***^	14.28 ± 0.09^b/***c***^	11.98 ± 0.12^c/***c***^	24.38 ± 0.00^a/***c***^	23.62 ± 0.15^ad/***c***^	19.55 ± 0.37^bc/***b***^	20.18 ± 1.67 ^cd/***b***^
**Propionic acid (mM)**								
**T0**	9.12 ± 0.51^a/***a***^	9.03 ± 0.38^a/***a***^	10.02 ± 1.03^a/***a***^	11.02 ± 0.13^a/***a***^	10.66 ± 0.64^a/***a***^	11.02 ± 0.13^a/***a***^	11.38 ± 2.44^a/***a***^	11.38 ± 2.18^a/***a***^
**T1**	26.35 ± 1.54^a/***b***^	24.44 ± 2.18^a/***b***^	25.80 ± 3.33^a/***b***^	25.35 ± 1.41^a/***b***^	33.24 ± 0.26^a/***b***^	31.97 ± 1.54^a***/b***^	26.35 ± 2.05 ^b/***b***^	35.06 ± 0.00^a/***b***^
**T2**	30.43 ± 1.15^a/***b***^	28.53 ± 0.77^a/***b***^	31.52 ± 0.64^a/***b***^	30.70 ± 1.54^a/***c***^	34.33 ± 1.80^a/***b***^	39.20 ± 1.84^a/***c***^	33.69 ± 1.67^a/***b***^	36.86 ± 3.32^a/***b***^
**Butyric acid (mM)**								
**T0**	1.87 ± 0.17^a/-^	1.97 ± 0.51^a/-^	2.06 ± 0.03^a/^*	1.92 ± 0.38^a/-^	3.73 ± 0.21^a/***a***^	3.76 ± 0.10^a/***a***^	3.56 ± 1.06^a/***a***^	4.00 ± 0.10^a/***a***^
**T1**	u.d.l. ^−/−^	u.d.l. ^−/−^	8.05 ± 0.43^-/^*	u.d.l. ^−/−^	12.76 ± 0.15^c/***b***^	7.48 ± 0.10^a/***b***^	9.52 ± 0.58 ^b/***b***^	13.27 ± 0.21^c/***b***^
**T2**	u.d.l. ^−/−^	u.d.l. ^−/−^	u.d.l. ^−/−^	u.d.l. ^−/−^	4.49 ± 0.10^c/***a***^	3.76 ± 0.17^a/***a***^	4.70 ± 0.07^ac/***a***^	6.34 ± 0.40^b/***c***^

One-way ANOVA and individual post hoc comparisons with the Tukey-Kramer was performed separately for both each sampling point and each bioreactor. For every colon tract (PC and DC), values in the same row with different regular superscript letters differ significantly (P < 0.05) (comparison among bioreactors of different 4-SHIME® units at the same sampling point), while values in the same column with different bold/italic superscript letters differ significantly (P < 0.05) (comparison in the same bioreactor at different sampling points). For butyric acid in PC3, values in the same column (comparison in the same bioreactor at different sampling points) with asterisks differ significantly according to Student's t-test (P < 0.05). The data are the means of three independent analysis ± standard deviations (n = 3). U.d.l. means under detection limit. “-“ means no determined.

### 3.6 Feeding with fermented juices reshapes colon microbiota

**Fig. 4:**
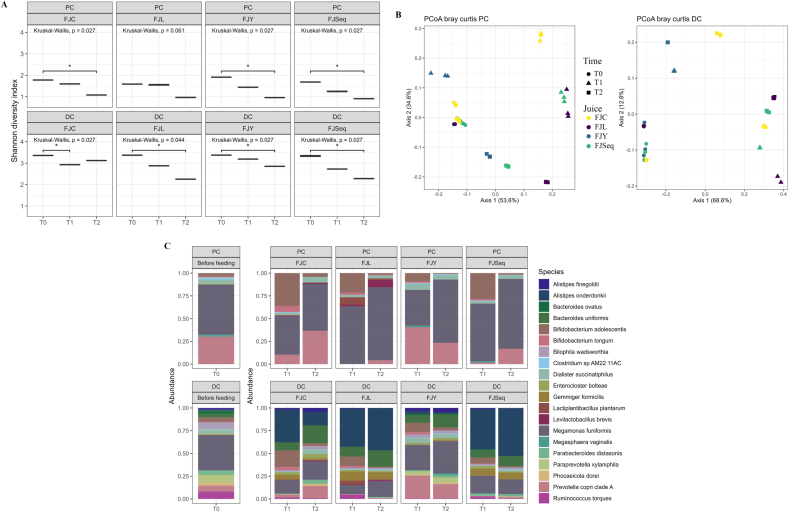
Shannon diversity index (A), relative abundance at species level of the bacterial microbiota before (T0), after one (T1) and two (T2) weeks of SHIME feeding with raw apple-pear juice (FJC), apple-pear juice fermented with *Apilactobacillus kunkeei* BEE4 for 96 h (FJL), apple-pear juice fermented with *Saccharomyces cerevisiae* KFAY2 for 72 h (FJY), and apple-pear juice fermented sequentially by *A. kunkeei* BEE4 for 72 h and then followed by *S. cerevisiae* KFAY2for 48 h (FJSeq), in proximal (PC) and distal (DC) colon reactors (B). Principal Coordinate Analysis (PCoA) of relative abundances of different species within the communities based on Bray–Curtis distance matrix (C).

The consumption of different juices affected the Shannon diversity index (Fig. 4A). After a two-week period, a general significant reduction (P < 0.05) in the diversity index was evident, reflecting a decline in microbial diversity following the feeding with all juices, except for FJL in PC, and FJC after two weeks in DC. Bray-Curtis dissimilarity metric was applied to compare the relative abundances of different species within the communities. Dissimilarities were visualized through the PCoA (Fig. 4B). Although the initial community structures were similar among SHIME units, the intake of juices modulated the composition within both the distal and proximal colon (P = 0.001).

**Fig. 5:**
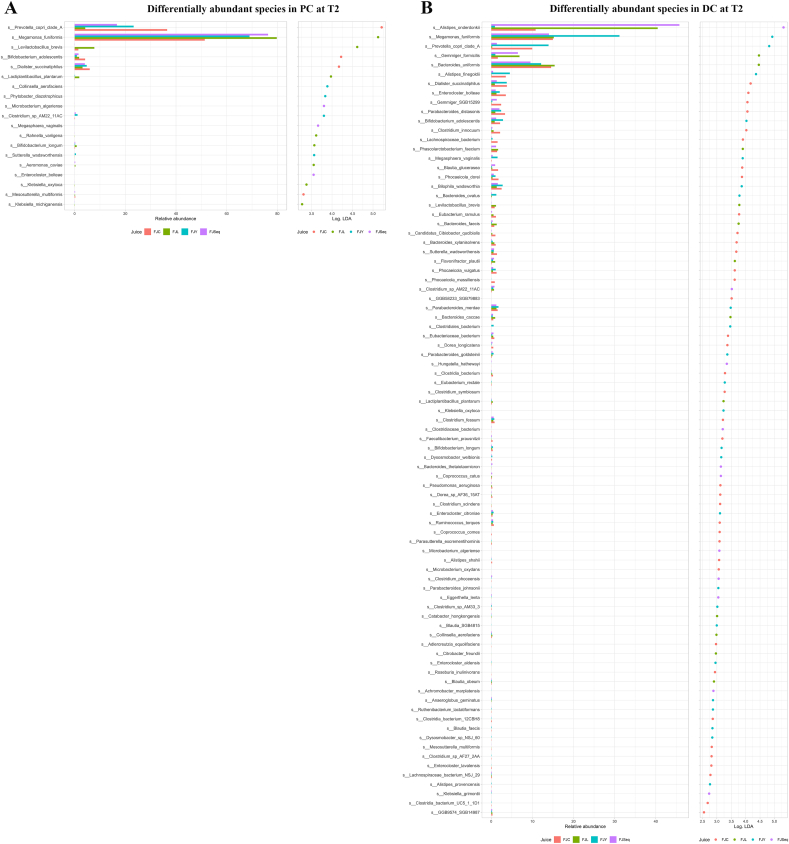
Differentially abundant species due to the intake of raw apple-pear juice (FJC), apple-pear juice fermented with *Apilactobacillus kunkeei* BEE4 for 96 h (FJL), apple-pear juice fermented with *Saccharomyces cerevisiae* KFAY2 for 72 h (FJY), apple-pear juice fermented sequentially by *A. kunkeei* BEE4 for 72 h and then followed by *S. cerevisiae* KFAY2for 48 h (FJSeq) in the proximal (panel a) and distal (panel b) colon tracts after 14 days of feeding (T2). For each panel, the left part shows the relative abundance of each species in each juice, while the right part shows the log LDA score of the comparison – the higher the value the higher the difference. The color of the LDA score shows which juice has the highest log mean abundance of each species. All adjusted p-values are <0.05. (For interpretation of the references to color in this figure legend, the reader is referred to the Web version of this article.)

Indeed, in PC, *Megamonas funiformis* and *Prevotella copri* were the highest abundant species (56. and 20.2%, respectively) at T0 in all SHIME lines. The intake of juices modulated the composition (Fig. 4C). Even if *M. funiformis* and *P. copri* remained the most abundant, an increase of *Bifidobacterium adolescentis* was found at T1, with FJC reaching the highest abundance, although the change did not persist at T2. The same pattern was found for *L. plantarum* after intake of FJL. Comparing PCs fed with different juices at the same timepoint, the linear discriminant analysis (LDA) effect size (LEfSe) showed that 22 and 19 species were differentially abundant (LDA >2, P < 0.05) after T1 and T2 of treatment, respectively (Fig. 5A). *L. plantarum* and *Levilactobacillus brevis* were higher after feeding with FJL. *Bifidobacterium longum* was more abundant in PC fed with FJC at T1, while at T2 the highest level was found in the PC fed with FJL*.* In DC, *M. funiformis* (28.8%), *Paraprevotella xylaniphila* (6.2%) and *Ruminococcus torques* (6.1%) were the most abundant species at T0, and the treatment differently modulated the composition depending on the juice. In detail, *Alistipes onderdonkii* became the most abundant species after one week (T1) of feeding with FJC, FJL and FJSeq (27.8, 34.8 and 26.7%, respectively), followed by *Bacteroides uniformis* (7.1, 6.6 and 6.5%), *B. adolescentis* (6.9, 5.2 and 13.6%) and *M. funiformis* (67, 15.1 and 11.0%). Differently, the intake of FJY maintained *M. funiformis* as the most abundant (21.7%), followed by *P. copri* (19.7%), *B. adolescentis* (8.0%) and *B. uniformis* (6.5%). The LDA LEfSe highlighted 95 and 85 differentially abundant (LDA >2, P < 0.05) species among DCs fed with different juices at T1 and T2, respectively (Fig. 5B). *L. plantarum* and *L. brevis* were more highly abundant after intake of FJL. Although, at T1, *B. adolescentis* and *B. longum* were more abundant after FJC intake, followed by FJY, FJL and FJSeq, at T2 they were both more highly abundant in the DC fed with FJY. *Faecalibacterium prausnitzii* was more highly abundant, at T1, in the DC fed with FJY and FJSeq, while at T2 it resulted more abundant after feeding with FJC followed by FSeq.

### 3.7 Feeding with fermented juices modulates gut microbiome

**Fig. 6:**
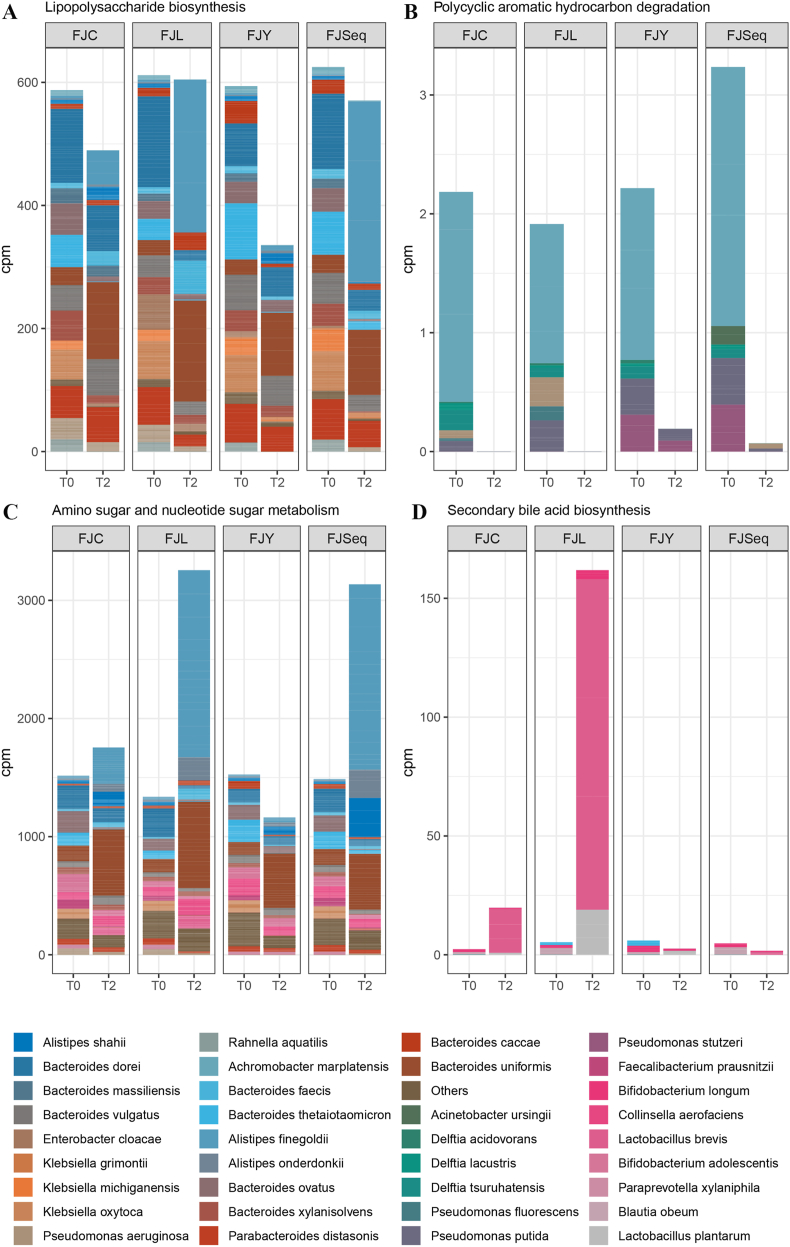
Pathways linked with lipopolysaccharide biosynthesis (A), polycyclic aromatic hydrocarbon degradation (B), amino sugar and nucleotide sugar metabolism (C), and secondary bile biosynthesis (D) before (T0) and after two (T2) weeks of feeding with raw apple-pear juice (FJC), apple-pear juice fermented with *Apilactobacillus kunkeei* BEE4 for 96 h (FJL), apple-pear juice fermented with *Saccharomyces cerevisiae* KFAY2 for 72 h (FJY), apple-pear juice fermented sequentially by *A. kunkeei* BEE4 for 72 h and then followed by *S. cerevisiae* KFAY2 for 48 h (FJSeq). Panels A, B and C refer to distal colon. Panes D refers to proximal colon.

A total of 4800 KEGG Orthologs (KO) were identified in the dataset, in 201 KEGG pathways related with energy, carbohydrates and other secondary metabolites synthesis, and with amino acids, lipids, cofactors and vitamins metabolisms were inspected (Table S3). The intake of fermented juices affected several pathways linked with obesity or weight loss as evident at the end of treatment period compared to T0 (Fig. 6). In PC, genes involved in secondary bile acids biosynthesis increased their abundance after intake of FJL, mainly due to the increase of *Lactobacillus* and *Bifidobacterium* relative abundances. In DC, FYJ consumption resulted in the highest decrease of genes related to lipopolysaccharide biosynthesis, followed by FJC and FJSeq. A reduction of microbial pathways linked to polycyclic aromatic hydrocarbon (PAH) degradation was found in DC after the intake of all juices. An increase of amino and nucleotide sugar metabolism pathway was also detected in DC microbiome after intake of FJL and FJSeq, mainly because of higher abundance of *Alistipes* and *Bacteroides.* After the intake of juices, the pathway leading to butyric acid synthesis resulted with lack of genes encoding for key enzymes in PC, while it was present in DC (Fig. S2).

### 3.8 Low sugar fermented juices exert a protective effect towards oxidative stress at intestinal level using *in vitro* models

**Fig. 7:**
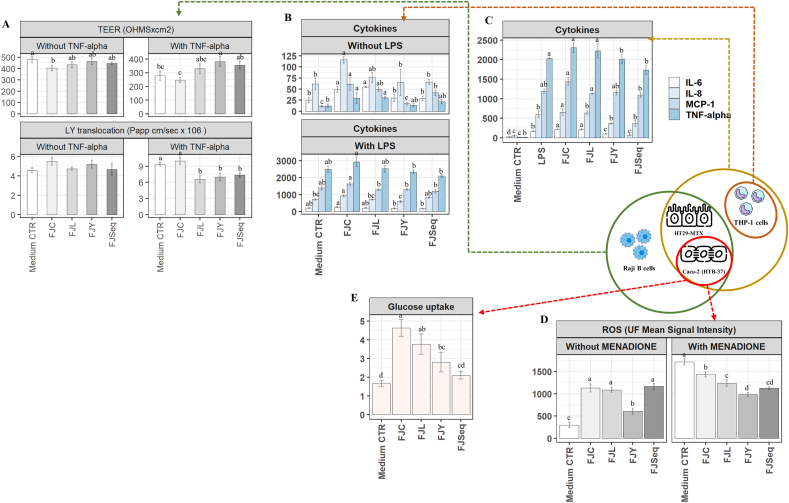
Transepithelial electric resistance and paracellular permeability of tri-culture cell layers (Caco-2/Ht-29 MTX/Raji B cells) (A), cytokine release from differentiated THP-1 macrophages (B) and tri-culture cell models consisting of Caco-2, HT29-MTX, and THP-1 cells (C), intracellular reactive oxygen species (ROS) (D) and glucose intake of Caco-2 cells (E). Cell culture models were treated with raw apple-pear juice (FJC), apple-pear juice fermented with *Apilactobacillus kunkeei* BEE4 for 96 h (FJL), apple-pear juice fermented with *Saccharomyces cerevisiae* KFAY2 for 72 h (FJY), apple-pear juice fermented sequentially by *A. kunkeei* BEE4 for 72 h and then followed by *S. cerevisiae* KFAY2for 48 h (FJSeq). Fermented apple-pear juices were incubated at 30 °C. Furthermore, to induce oxidative stress cell cultures were treated with TNF-alfa, LPS, and menadione. Data points with different superscript letters (a–d) differ significantly (P < 0.05).

Upon TNF-alpha exposure, only fermented juices revealed enhancements in the integrity and barrier function of tri-culture cell layers (Caco-2/Ht-29 MTX/Raji B cells), demonstrated by elevated TEER and reduced paracellular permeability (P < 0.05). Cytokine release from differentiated THP-1 macrophages in absence of LPS stimulation indicated that FJC and FJL had notably higher IL-6 and TNF α levels compared to the control medium. Conversely, only FJC showed higher values of IL-8 than the control medium and only FJY exhibited comparable MCP-1 levels. With LPS, all treatments led to increased cytokine release, with FJC showing consistently higher significant levels compared to fermented juices (P < 0.05). Under the same experimental conditions, the focus was shifted towards stimulated tri-culture cell models consisting of Caco-2, HT29-MTX, and THP-1 cells. In the presence of LPS, FJC and FJL showed the highest level of IL-6 secretions when compared to the LPS-stimulated group, whereas FJSeq and FJY displayed an opposing statistically significant trend (P < 0.05). The same former samples (FJC and FJL) demonstrated the highest levels of TNF α and IL-8 secretion. Conversely, FJSeq and FJY caused significantly lower values of IL-8 when compared to FJC, whereas only FJSeq demonstrated a significant deviation in TNF α secretion. Turning attention to MCP-1, FJC consistently exhibited the highest significant values and FJSeq the lowest. ROS, a marker of oxidative stress, markedly increased after Menadione application. The detrimental impact of Menadione was significantly mitigated (P < 0.05) by fermented juices and, particularly with FJY. Radiolabeled glucose analogs noted the highest significant glucose intake was observed in wells of Caco-2 cells supplemented with FJC, followed by FJL, FJY, and FJSeq, when compared to the medium CTR (Fig. 7).

### 3.9 Sensory analysis

On a ten-point scale, seven sensory attributes were evaluated (Table S4). Panelist rated the highest significant (P < 0.05) sweetness score to FJC, followed by FJL, FJSeq, and FJY. In terms of acidity, FJL received the highest score, with FJSeq ranking next, while FJY and FJC exhibited the lowest scores. Flavor intensity was most pronounced (P < 0.05) in FJL, whereas FJY and FJSeq showed the lowest scores. Remaining attributes and overall rating did not show significant (P > 0.05) variation across the different samples.

## 4 Discussion

The goal of reducing sugar consumption and calorie intake, especially in children and adolescents, is an important challenge to ensure a healthy lifestyle. Considering that fruit-based juices are a convenient alternative to the consumption of fresh fruits but at the same time are a significant source of sugar, we developed new fermented fruit juices with lower sugars content but higher levels of low-calorie sweetener and good sensory ratings. Apple-pear juice was used as model system.

The multifaceted nature of fermentation allows to redesign food functionality in a versatile way, based on involved microorganisms and specific conditions employed (Tlais et al., 2022). The selection criteria for microorganisms were rooted in their ability to hydrolyze predominant sugars in synthetic and juice-based media, mirroring the target substrates. Among LAB candidates, we considered also the capacity to produce mannitol, due to its nature of low-calorie sweetener (Jeon et al., 2021). Given considerations of labor and metabolic specialization, we selected *A. kunkeei* BEE4 and *S. cerevisiae* KFAY2 as cultures for directing fermentation in three distinct trajectories, involving them both as monoculture and sequential with yeasts preceding LAB. Under our experimental conditions, these microorganisms played a pivotal role reducing 27% (FJL), 54% (FJSeq), and 66% (FJY) of sugar content within a challenging ecological niche such as apple and pears, concurrently elevating mannitol levels.

The cause-effect relationships between the intake of new fermented juices and the evolution of gut microbiota were explored to verify the impact of low sugar fermented juices on the colon ecosystems. To this purpose, the SHIME *in vitro* model was used to mimic *in vivo* conditions eliminating potential interferences from dietary factors and human physiology (Da Ros et al., 2021). The selection of the fecal donor is basic to evolve representative gut ecosystems (Polo et al., 202). After the colon bioreactors reached stable microbial ecosystems, each 4-SHIME unit was fed with 200 mL/day of apple-pear juices for 14 days, according to dietary recommendations (Ruxton and Myers, 2021). Compared to control juice (without microbial inoculum), feeding with fermented juices reshaped the gut microbiota, and several beneficial taxa increased their abundances. Lactic acid fermented juice (FJL) promoted *L. plantarum* and *L. brevis* which, overall, were associated with good adaptation and adhesion ability in the gut ecosystems, and with probiotic, activities in both *in vitro* and *in vivo* studies (Garcia-Gonzalez et al., 2021; Somashekaraiah et al., 2021). The administration of probiotics containing lactobacilli strains to obese animals was reported to effectively reduce body fat mass and to improve both lipid distribution and blood glucose homeostasis (Geng et al., 2022). Despite a short-term increase after feeding with the control FJC, *B. longum* displayed the highest growth in the longer-term period in the PC fed with FJL. This species is characterized by cholesterol reduction and mucin adhesion-related genes and has been associated with several health-promoting effects, as demonstrated both *in vitro* and *in vivo* (Kim and Kim, 2023; Mandelbaum et al., 2023). The higher abundance of *B. adolescentis* and *B. longum* in DC after longer-term intake of juice fermented with yeast (FJY) represents also a promising ecological evolution, since both species are producer of the neuro-transmitter gamma aminobutyric acid (Duranti et al., 2020). Moreover, *B. adolescentis* is known for alleviating symptoms of type 2 diabetes by restoring the homeostasis of the gut microbiota, increasing the abundance of short-chain fatty acid-producing microbiota (Qian et al., 2022). More in general, beneficial microorganisms like lactobacilli and *Bifidobacterium* spp. have been demonstrated to be a promoting measure to combat obesity and related diseases (Geng et al., 2022). In DC, the intake of FJC and the juice fermented with sequential use of microorganisms (FJSeq) reflected in higher abundance of *F. Prausnitzii*, a well-known health promoting species with anti-inflammatory properties and capacity to enhance the intestinal barrier function and affect paracellular permeability (Lopez-Siles et al., 2017). It is also a promising biomarker for assisting diagnosis and prognostic of gut diseases such as ulcerative colitis and Crohn's disease (Lopez-Siles et al., 2017), while such genus notably decreased in obese compared to normal-weight people (Geng et al., 2022). Although some authors consider *Meganomonas* species harmful bacteria (Zhu et al., 2024), some strains of *M. funiformis*, which increased in both PC after intake of all fermented juices (FJL > FJSeq > FJY > FJC) and DC after consumption of FJY, were suggested as probiotic candidates for preventative or therapeutic intervention of metabolic dysfunction-associated fatty liver disease (Yang et al., 2023). The role of *Alistipes*, which became more abundant in DC after feeding with fermented juices, is also still debated as it may depend on the host, and the bodily system. In terms of pathogenicity, a protective effect against several diseases was hypothesized (Parker et al., 2020). An enrichment of *Alistipes* was reported in obese individuals that consistently succeeded in losing their weight compared to patients who were less successful in weight reduction (Louis et al., 2016), while a decrease has been observed in obese compared to normal-weight people (Geng et al., 2022). Specifically, *A. onderdonkii* increased after the intake of FJSeq and FJL, while its reduction in the gut microbiome was recently considered an advantage for the proliferation for pancreatic cancer cells (Lee et al., 2021). On the contrary, *A. finegoldii* reached the highest abundances after feeding with FJY and FJC, and it was associated with a protective effect against colitis, but also with systolic blood pressure and intestinal inflammation (Parker et al., 2020). *P. copri* is a common intestinal species mainly associated with plant-based diets. Although in PC it reached the highest abundance after feeding with no fermented FJC, in DC it was higher after the intake of FJY. Based on the prevalence of carbohydrate metabolism genes in such species, it can be hypothesized that its growth in PC was stimulated by the higher sugars content of FJC, while in the DC, where lumen sugars are mostly already depleted, the highest abundance corresponded to FJY feeding. In general, *P. copri* has been associated with both positive and negative impacts on diseases such as Parkinson's disease and rheumatoid arthritis, and the debate on its role in human health is still open (Abdelsalam et al., 2023). *Gemmiger formicilis,* which had higher abundances in DC after the intake of FJL and FJSeq, is known for its high SCFA production (Liu et al., 2023). *Bacteroides uniformis,* which became more abundant in DC after feeding with FJL, was associated in animal studies with intestinal homeostasis, regulation of bile acid metabolism, proliferation of potentially beneficial bacteria, reduction of serum cholesterol, triglyceride, glucose and insulin levels in obese subjects, and alleviating immune disorders (Yan et al., 2023).

The reshape of colon microbiota reflected on the SCFAs content in colon tracts. SCFAs play an important role in gut ecosystem wellbeing regulating for instance glucose and lipids metabolism (de Carvalho et al., 2021). The intake of all juices produced higher concentrations of acetic and propionic acids in both PC and DC. In particular, in PC tract, the juices consumption prompted a temporary increase of acetic acid in the short-term period (1 week) coherently with the microbiota modulation, which also highlighted an evolution from the first to the second week of intake. The partial acetic acid decrease observed during the second week of juices feeding was consistent with previous studies that stated a transient nature of diet-induced changes in SCFA content. Indeed, despite a fast reaction to a diet intervention is observed, often the acetic acid does not remain at the highest level but drops even if the intervention continues, and it reaches an equilibrium after a gradual stabilization (Liu et al., 2022). Acetic acid by colonic fermentation, which reached the highest level after feeding with no fermented juice in PC and after intake of FJL and FJC in DC, is known to cross the blood–brain barrier and act as an appetite suppressant in hypothalamus, thus suggesting a potentially relevant role in the management of metabolic diseases including obesity and diabetes (Frost et al., 2014). Considering that the daily dose was the same for the different juices (200 mL/day), the highest levels associated to FJC and FJL may be due to the specific composition of such juices, since chemical inputs combined with the metabolic capacity of involved species to use such substrates for growing are the most important drivers for the ecological evolution of a microbial population (De Paepe et al., 2020). Indeed, the new feeding regime shaped the microbial communities favoring species with metabolic capacity to use and efficiently assimilate the different substrates, addressing microbiota evolution and its metabolic answer. For instance, in PC this ecological dynamic resulted in the increase in relative abundance of *P. copri*, that is able to use glucose highly abundant in FJC, with the consequent enhancement of acetic acid concentration (Huang et al., 2021). Similarly, it can be hypothesized that *B. uniformis* and *G. formicilis* increased in DC likely thanks to chemical characteristics of FJC and FJL. Indeed, enhancing glucose utilization, they induce a higher acetate production through carbohydrate metabolism (López-Almela et al., 2021; Liu et al., 2023). Overall, these findings demonstrate anyway a potentially beneficial enhancement of acetic acid synthesis at colon level compared to T0 due to the feeding with fermented juices (specially FJL) despite they correspond to a lower sugar intake compared to FJC. Similarly, also the level of propionic acid did not differ based on different juices. On the contrary, butyric acid disappeared in PC after the intake of both fermented and no fermented juices, while in DC it had a permanent increase only corresponding to FJSeq feeding. Such finding is coherent with metagenomic results which demonstrated the lack of genes encoding for key enzymes in the butanoate metabolism pathway in PC after the feeding with juices, while the pathway was present in the microbiome of DC. This can be explained by the very low abundance of main butyrate producers like *F. prausnitzii, Bacteroidetes* and *Clostridia* (Lopez-Siles et al., 2017; Parada Venegas et al., 2019).

The reshape of microbiota reflected on several microbiome functional changes linked with obesity or weight loss. In PC, the increased abundance of genes involved in biosynthesis the secondary bile acids after feeding with FJL (most likely as a consequence of the increased relative abundance of *Lactobacillus* and *Bifidobacterium*) was found. Indeed, they are the end products of cholesterol metabolism, and they play an important role in dietary fat digestion and absorption. Moreover, being signal molecules, they modulate host lipid, energy and glucose/insulin metabolism, and inflammation (Geng et al., 2022). The decreased abundance of genes related to lipopolysaccharide biosynthesis observed in DC after the intake of FYJ and, to a lesser degree, of FJC and FJSeq was another significant finding. In fact, it was hypothesized that bacterial lipopolysaccharide from bacteria inhabiting the gut may be the trigger for increased inflammation in colon ecosystems, which is considered a basic condition in the metabolic processes leading to obesity, metabolic syndrome, and diabetes (Devaraj et al., 2013). Also, the reduction of microbial pathways linked to PAH degradation, found in DC after the intake of all juices, can be read as a promising effect since these pathways (yielding to obesogenic compounds produced during the incomplete combustion of organic matter) are overrepresented in individuals that are unable to lose weight (Louis et al., 2016). Changes in the abundance of amino and nucleotide sugar metabolism pathways, that we found increased in DC microbiome after intake of FJL and FJSeq, were already reported in obese individuals during a weight loss therapy (Louis et al., 2016). In our case study, it can be linked to the increase abundance of *Alistipes* and *Bacteroides.*

While dietary components possess the capacity to incite intestinal inflammation and modify both mucosal integrity and permeability, empirical evidence in this domain remains limited (Guibourdenche et al., 2021), and little is known about the effects of direct exposure to low sugar fermented juices on the intestinal barrier and on the cellular mechanisms they may alter. Low sugar intake appears to bolster epithelial barrier function by influencing tight junction proteins, thereby increasing TEER and reducing paracellular permeability. The breakdown of the intestinal mucosal barrier plays a substantial role in the development of type 2 diabetes (T2D), (Horton et al., 2014; Amin et al., 2018). High sugar intake generates ROS through glucose oxidation and protein glycosylation, disrupting the balance between antioxidants and oxidants, altering permeability in epithelial cell layers, potentially contributing to obesity and diabetes complications (Sharma et al., 2020). We and others have shown that sugary treatment triggered oxidative stress, elevating the secretion of pro-inflammatory cytokines (Sharma et al., 2020). The concurrent rise in IFN-α, IL-6, IL-8, and MCP-1 cytokine levels appears to correspond with heightened ROS production within culture cells. Therefore, modulation of cytokines levels by low sugar fermented juices serves to attenuate oxidative damage severity within epithelial cells. Prior study has posited a link between morbid obesity and increased intestinal glucose absorption, possibly stemming from irregularities in the intestinal sweet taste receptors or glucose transporters (Nguyen et al., 2015). Thereafter, elevated intestinal glucose absorption due to control juice may trigger incretin responses, contributing to the observed manifestations of hyperglycemia, hyperinsulinemia, insulin resistance, and increased lipogenesis characteristic of obesity (Girard and Lafontan, 2008; Spector and Shikora, 2010).

## 5 Conclusions

This study presents novel fermented fruit juices having reduced sugar and caloric contents and good sensory ratings compared to traditional no-fermented counterpart. Their intake has been shown to orchestrate, *in vitro*, promising changes in the gut microbiota, its metabolic answer and functionality. Discernible functional changes within the microbiome, notably those associated with obesity or weight loss, are predominantly attributed to the specific influence of fermentation. Concurrently, the fermented juices showed positive modulation of intestinal epithelial features. Although the simulation used in the study simplifies the *in vivo* continuous and variable dietary influx, and the host conditions, our study suggests that the low sugar level of fermented juices activates a targeting gut ecosystem answer.

## Funding

This work was supported by the European Regional Development Fund (ERDF) 2014–2020 of the Autonomous 10.13039/501100015273Province of Bolzano (FESR1149 SMARTJUICE project). The APC was funded by the Open Access Publishing Fund of the 10.13039/501100008815Free University of Bozen-Bolzano.

## Ethical statement

This study was approved by the Ethics Committee of the Free University of Bozen on July 17, 2019.

The datasets used and/or analyzed during the current study are available from the corresponding author upon request.

## CRediT authorship contribution statement

**Ali Zein Alabiden Tlais:** Methodology, Investigation, Data curation, Formal analysis, Writing – original draft. **Andrea Polo:** Conceptualization, Methodology, Investigation, Data curation, Formal analysis, Writing – original draft. **Lena Granehäll:** Data curation, Formal analysis, Software. **Pasquale Filannino:** Writing – review & editing. **Olimpia Vincentini:** Investigation, Formal analysis. **Francesca De Battistis:** Investigation, Formal analysis. **Raffaella Di Cagno:** Conceptualization, Methodology, Supervision, Writing – review & editing. **Marco Gobbetti:** Funding acquisition, Project administration, Supervision, Writing – review & editing.

## Declaration of competing interest

The authors declare that they have no known competing financial interests or personal relationships that could have appeared to influence the work reported in this paper.

## Data Availability

No data was used for the research described in the article.
