# Supplementary material for: Sugar lowering in fermented apple-pear juice orchestrates a promising metabolic answer in the gut microbiome and intestinal integrity
Source: Curr Res Food Sci. 2024 Sep 5;9:100833. doi: 10.1016/j.crfs.2024.100833 (PMC11406026; doi:10.1016/j.crfs.2024.100833)

**Figure S1.** Schematic representation of the apple-pear juices fermentation methods adopted in this study.


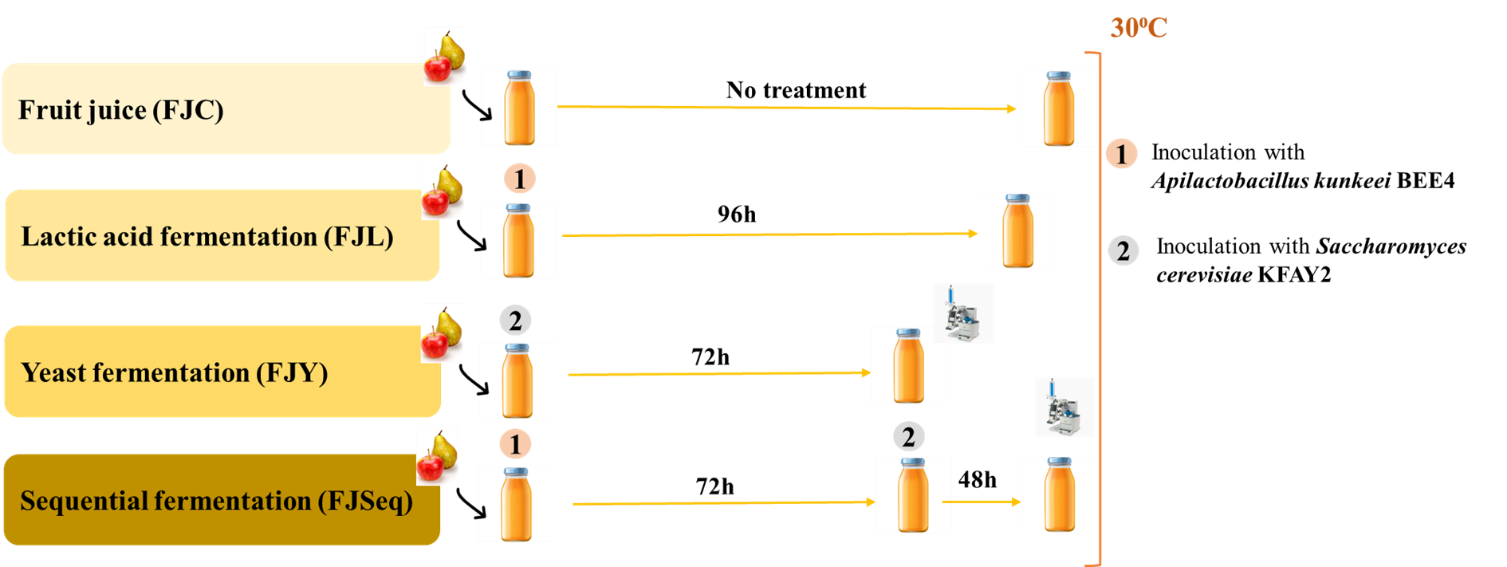

Supplement: Multimedia component 1 [file mmc1.docx]
