# Supplementary material for: Sugar lowering in fermented apple-pear juice orchestrates a promising metabolic answer in the gut microbiome and intestinal integrity
Source: Curr Res Food Sci. 2024 Sep 5;9:100833. doi: 10.1016/j.crfs.2024.100833 (PMC11406026; doi:10.1016/j.crfs.2024.100833)

**Figure S2.** Overview of butyric acid synthesis pathway (Kegg pathway 00650) in proximal colon (PC) (A) and distal colon (DC) (B) microbiome. Filled rectangles along the pathway represent the presence/abundance of key enzymes after 14 days of feeding (T2) with the four different juices (order within the rectangles: FJC, FJL, FJY, FJSeq). The color within the rectangles reppresents the values, according to the color scale, reported as logarithmic CPM (copies per million). In both panels, the empty rectangle with red border highlights the enzymes in the last step of the butyric acid synthesis (K00929/2.7.2.7, K01034, K01035 and K19709/2.8.3.8), that are present in DC but absent in PC.


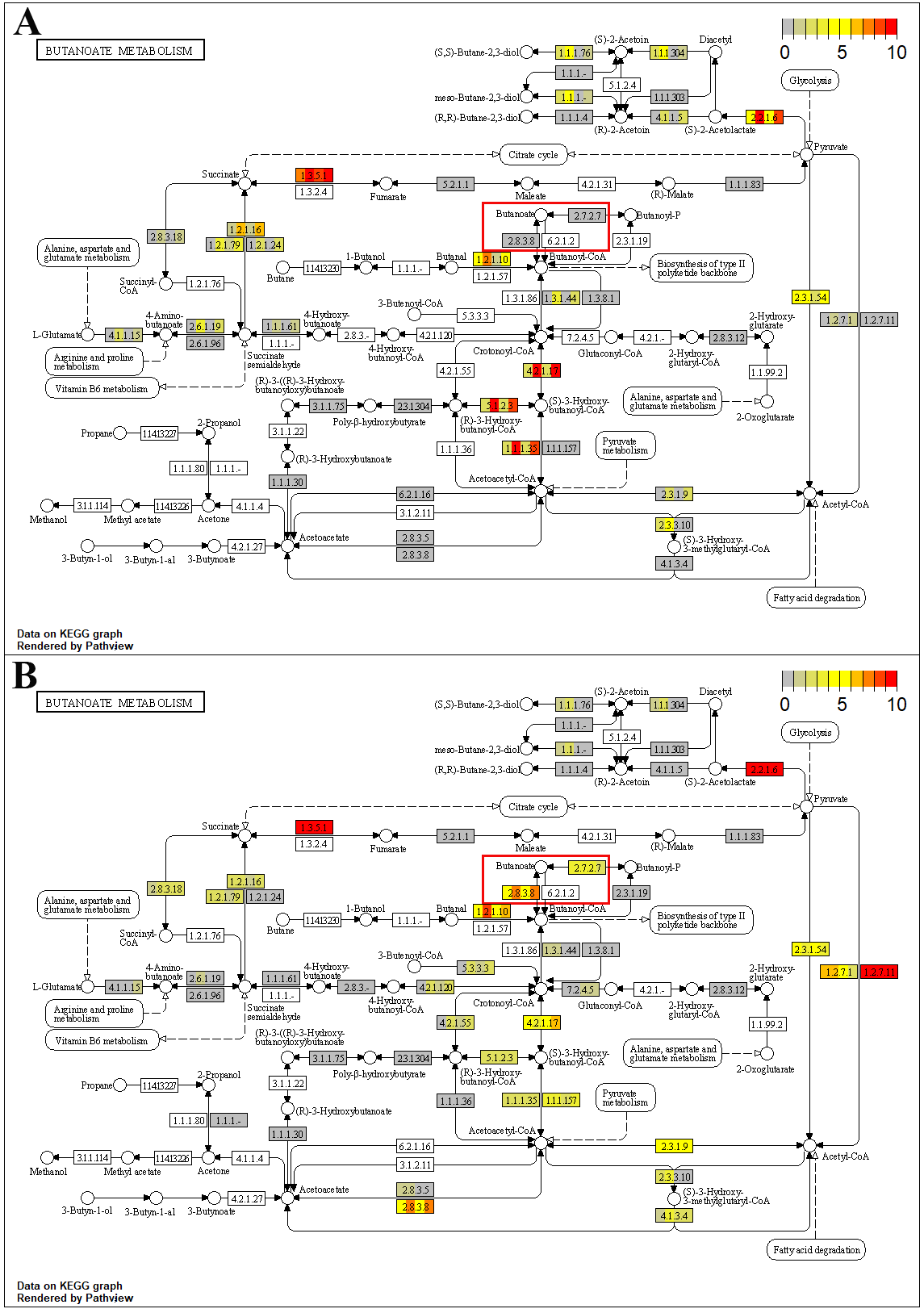

Supplement: Multimedia component 2 [file mmc2.docx]
