# Supplementary material for: Sugar lowering in fermented apple-pear juice orchestrates a promising metabolic answer in the gut microbiome and intestinal integrity
Source: Curr Res Food Sci. 2024 Sep 5;9:100833. doi: 10.1016/j.crfs.2024.100833 (PMC11406026; doi:10.1016/j.crfs.2024.100833)
Supplement: Multimedia component 3 [file mmc3.docx]

**Table S1.** List of lactic acid bacteria and yeasts used in this study.

| Species | Strains |
| --- | --- |
| Lactic acid bacteria |  |
| *Apilactobacillus kunkeei* | PL13, BEE4, BV61 |
| *Companilactobacillus paralimentarius* | RL3 |
| *Enterococcus durans* | JKmL2, JKmL3, KfBL2, KfBL3, KmL6, KmL7 |
| *Enterococcus faecium* | KfBL1 |
| *Fructilactibacillus sanfranciscensis* | RL2 |
| *Fructobacillus fructosus* | PL10, PL25, PL22 |
| *Lacticaseibacillus paracasei* | KfAL1, KfAL2 |
| *Lactiplantibacillus fabifermantans* | ALII9 |
| *Lactiplantibacillus plantarum* | EL1, EL2, GL5, GL7, GSL3, KmL4, KmL3, SL6, SL7, S6w5 |
| *Leuconostoc mesenteroides* | GL1, GL2, GSL1, GSL2, SL1, SL2, S3d1 |
| *Pediococcus parvulus* | S4w10 |
| Yeasts |  |
| *Saccharomyces cerevisiae* | AN6Y1O, KFAY2, RY1, RY2 |
| *Clavispora lusitaniae* | GY3, KFAY4 |
| *Kazachstania unispora* | KFBY1 |
| *Hanseniaspora uvarum* | SY1 |
| *Pichia manshurica/membranifaciens* | KTB6Y1, KTS6C1 |
| *Zygosaccharomyces bailii/parabailii* | KTB9C2, KTB6C1 |
| *Brettanomyces anomalus* | JB15G1, KS12C1 |
| *Zygosaccharomyces bisporus* | JB9G2, JB6Y2 |
| *Schizosaccharomyces pombe* | JB151G1 |
| *Schizosaccharomyces osmophilus/pombe* | JB6C2 |
