# Supplementary material for: Sugar lowering in fermented apple-pear juice orchestrates a promising metabolic answer in the gut microbiome and intestinal integrity
Source: Curr Res Food Sci. 2024 Sep 5;9:100833. doi: 10.1016/j.crfs.2024.100833 (PMC11406026; doi:10.1016/j.crfs.2024.100833)
Supplement: Multimedia component 4 [file mmc4.docx]

**Table S2**. Fecal microbiota composition of selected donor at genus level.

| **Kingdom** | **Phylum** | **Class** | **Order** | **Family** | **Genus** | **Abundance** |
| --- | --- | --- | --- | --- | --- | --- |
| Bacteria | Firmicutes | Clostridia | Oscillospirales | Ruminococcaceae | Faecalibacterium | 46,46668 |
| Bacteria | Firmicutes | Clostridia | Lachnospirales | Lachnospiraceae | Blautia | 11,50456 |
| Bacteria | Firmicutes | Clostridia | Lachnospirales | Lachnospiraceae | Coprococcus | 9,185094 |
| Bacteria | Firmicutes | Clostridia | Lachnospirales | Lachnospiraceae | [Eubacterium] hallii group | 3,533323 |
| Bacteria | Firmicutes | Clostridia | Oscillospirales | Ruminococcaceae | Subdoligranulum | 3,425081 |
| Bacteria | Firmicutes | Clostridia | Lachnospirales | Lachnospiraceae | Fusicatenibacter | 2,845214 |
| Bacteria | Firmicutes | Clostridia | Lachnospirales | Lachnospiraceae | Roseburia | 2,752435 |
| Bacteria | Firmicutes | Clostridia | Lachnospirales | Lachnospiraceae | [Ruminococcus] torques group | 2,28081 |
| Bacteria | Firmicutes | Clostridia | Lachnospirales | Lachnospiraceae | Anaerostipes | 1,654554 |
| Bacteria | Firmicutes | Clostridia | Lachnospirales | Lachnospiraceae | [Ruminococcus] gauvreauii group | 1,461265 |
| Bacteria | Bacteroidota | Bacteroidia | Bacteroidales | Prevotellaceae | Prevotella_9 | 1,430339 |
| Bacteria | Firmicutes | Clostridia | Lachnospirales | Lachnospiraceae | [Eubacterium] ruminantium group | 1,383949 |
| Bacteria | Firmicutes | Clostridia | Lachnospirales | Lachnospiraceae | Dorea | 1,329828 |
| Bacteria | Firmicutes | Clostridia | Oscillospirales | Ruminococcaceae | Ruminococcus | 1,283439 |
| Bacteria | Firmicutes | Clostridia | Oscillospirales | Oscillospiraceae | UCG-002 | 1,167466 |
| Bacteria | Firmicutes | Negativicutes | Veillonellales-Selenomonadales | Veillonellaceae | Dialister | 1,036029 |
| Bacteria | Bacteroidota | Bacteroidia | Bacteroidales | Bacteroidaceae | Bacteroides | 0,950982 |
| Bacteria | Firmicutes | Clostridia | Lachnospirales | Lachnospiraceae | CAG-56 | 0,873666 |
| Bacteria | Firmicutes | Bacilli | Erysipelotrichales | Erysipelatoclostridiaceae | Erysipelotrichaceae UCG-003 | 0,84274 |
| Bacteria | Firmicutes | Clostridia | Christensenellales | Christensenellaceae | Christensenellaceae R-7 group | 0,487088 |
| Bacteria | Firmicutes | Clostridia | Lachnospirales | Lachnospiraceae | Lachnospiraceae FCS020 group | 0,39431 |
| Bacteria | Bacteroidota | Bacteroidia | Bacteroidales | Barnesiellaceae | Barnesiella | 0,363383 |
| Bacteria | Firmicutes | Clostridia | Lachnospirales | Lachnospiraceae | Lachnospiraceae NK4A136 group | 0,355652 |
| Bacteria | Firmicutes | Clostridia | Lachnospirales | Lachnospiraceae | Marvinbryantia | 0,34792 |
| Bacteria | Firmicutes | Clostridia | Lachnospirales | Lachnospiraceae | [Eubacterium] ventriosum group | 0,316994 |
| Bacteria | Firmicutes | Clostridia | Lachnospirales | Lachnospiraceae | [Eubacterium] eligens group | 0,286068 |
| Bacteria | Bacteroidota | Bacteroidia | Bacteroidales | Prevotellaceae | Prevotellaceae NK3B31 group | 0,270605 |
| Bacteria | Bacteroidota | Bacteroidia | Bacteroidales | Rikenellaceae | Alistipes | 0,224215 |
| Bacteria | Firmicutes | Clostridia | Oscillospirales | Ruminococcaceae | [Eubacterium] siraeum group | 0,208752 |
| Bacteria | Firmicutes | Clostridia | Lachnospirales | Lachnospiraceae | Lachnospiraceae UCG-010 | 0,208752 |
| Bacteria | Firmicutes | Clostridia | Oscillospirales | Butyricicoccaceae | Butyricicoccus | 0,208752 |
| Bacteria | Firmicutes | Clostridia | Monoglobales | Monoglobaceae | Monoglobus | 0,177826 |
| Bacteria | Firmicutes | Clostridia | Oscillospirales | Ruminococcaceae | Incertae Sedis | 0,1469 |
| Bacteria | Bacteroidota | Bacteroidia | Bacteroidales | Tannerellaceae | Parabacteroides | 0,1469 |
| Bacteria | Actinobacteriota | Coriobacteriia | Coriobacteriales | Coriobacteriaceae | Collinsella | 0,115973 |
| Bacteria | Bacteroidota | Bacteroidia | Bacteroidales | Prevotellaceae | Alloprevotella | 0,092779 |
| Bacteria | Firmicutes | Clostridia | Oscillospirales | Oscillospiraceae | UCG-003 | 0,077316 |
| Bacteria | Firmicutes | Clostridia | Oscillospirales | Oscillospiraceae | NK4A214 group | 0,046389 |
| Bacteria | Firmicutes | Clostridia | Lachnospirales | Lachnospiraceae | [Eubacterium] xylanophilum group | 0,046389 |
| Bacteria | Proteobacteria | Gammaproteobacteria | Burkholderiales | Sutterellaceae | Sutterella | 0,046389 |
| Bacteria | Firmicutes | Clostridia | Peptostreptococcales-Tissierellales | Anaerovoracaceae | [Eubacterium] brachy group | 0,023195 |
