# Supplementary material for: Sugar lowering in fermented apple-pear juice orchestrates a promising metabolic answer in the gut microbiome and intestinal integrity
Source: Curr Res Food Sci. 2024 Sep 5;9:100833. doi: 10.1016/j.crfs.2024.100833 (PMC11406026; doi:10.1016/j.crfs.2024.100833)
Supplement: Multimedia component 5 [file mmc5.docx]

**Table S3.** Pathways (cpm) related with energy, carbohydrates and other secondary metabolites synthesis, and with amino acid, lipid, cofactors and vitamins metabolisms inspected in the study. PC and DC mean proximal and distal colon, respectively; FJC, FJL, FJY and FJSeq represent the different juices; T0, T1 and T2 mean before, and after one and two weeks of juice intake, respectively.

| **Kegg pathway** | **Kegg process** | **PC** | | | | | | | | | | | | **DC** | | | | | | | | | | | |
| --- | --- | --- | --- | --- | --- | --- | --- | --- | --- | --- | --- | --- | --- | --- | --- | --- | --- | --- | --- | --- | --- | --- | --- | --- | --- |
|  |  | **FJC** | | | **FJL** | | | **FJY** | | | **FJSeq** | | | **FJC** | | | **FJL** | | | **FJY** | | | **FJSeq** | | |
|  |  | **T0** | **T1** | **T2** | **T0** | **T1** | **T2** | **T0** | **T1** | **T2** | **T0** | **T1** | **T2** | **T0** | **T1** | **T2** | **T0** | **T1** | **T2** | **T0** | **T1** | **T2** | **T0** | **T1** | **T2** |
| 00010 Glycolysis / Gluconeogenesis | Carbohydrate metabolism | 6,525563 | 16,22026 | 1,452576 | 5,622932 | 20,3604 | 7,493198 | 7,02438 | 3,391457 | 0,48423 | 7,034443 | 16,4559 | 1,72866 | 10,3401 | 14,89428 | 9,864341 | 8,709834 | 15,18982 | 14,75175 | 9,824572 | 7,642502 | 7,318436 | 10,0643 | 14,4206 | 13,84918 |
| 00020 Citrate cycle (TCA cycle) | Carbohydrate metabolism | 4,770235 | 3,606585 | 0,337455 | 4,875568 | 10,91554 | 2,804111 | 5,870915 | 1,147858 | 0,145047 | 5,610233 | 6,686726 | 1,203582 | 6,472131 | 7,929859 | 7,265755 | 6,325298 | 8,917342 | 11,15196 | 6,029548 | 4,642177 | 4,969023 | 6,51928 | 9,705952 | 10,9163 |
| 00030 Pentose phosphate pathway | Carbohydrate metabolism | 6,964722 | 16,81102 | 1,826693 | 6,024041 | 19,29493 | 6,537858 | 7,38364 | 3,89696 | 0,654924 | 7,373487 | 17,48437 | 1,846112 | 10,8551 | 14,52778 | 9,45792 | 9,069542 | 14,63829 | 13,08885 | 10,49543 | 7,957189 | 7,061596 | 10,68695 | 13,48477 | 12,35158 |
| 00040 Pentose and glucuronate interconversions | Carbohydrate metabolism | 6,83129 | 8,974516 | 2,442473 | 6,112486 | 12,51084 | 6,405106 | 6,760638 | 3,581802 | 1,309043 | 7,619985 | 11,64224 | 2,068948 | 9,162543 | 6,098261 | 6,021808 | 7,590486 | 7,382655 | 5,339876 | 8,514619 | 6,407507 | 5,748386 | 8,870522 | 5,194259 | 3,772006 |
| 00051 Fructose and mannose metabolism | Carbohydrate metabolism | 4,493772 | 9,025604 | 0,415278 | 4,230233 | 12,41381 | 3,510957 | 5,047295 | 1,6215 | 0,227298 | 5,258171 | 8,57924 | 1,013038 | 6,646631 | 8,729246 | 6,009019 | 5,913533 | 8,627474 | 7,963689 | 6,544569 | 4,591577 | 4,80509 | 6,675248 | 7,536549 | 6,873443 |
| 00052 Galactose metabolism | Carbohydrate metabolism | 4,088575 | 15,65698 | 1,324668 | 3,645913 | 11,86649 | 5,471949 | 4,462538 | 2,425515 | 0,330019 | 4,501843 | 11,36329 | 1,078751 | 9,461864 | 15,37381 | 7,644692 | 7,429866 | 12,8637 | 11,39623 | 9,313853 | 6,733837 | 5,271433 | 9,431196 | 12,46878 | 11,0857 |
| 00053 Ascorbate and aldarate metabolism | Carbohydrate metabolism | 5,960181 | 5,534733 | 2,373476 | 5,68999 | 9,069884 | 3,23933 | 5,894884 | 3,369465 | 1,630214 | 6,803286 | 7,435015 | 1,981417 | 2,994443 | 2,710351 | 1,824809 | 2,756643 | 2,894087 | 0,934874 | 3,01017 | 3,258464 | 2,104002 | 3,012508 | 1,449129 | 0,740681 |
| 00061 Fatty acid biosynthesis | Lipid metabolism | 7,847901 | 14,8233 | 1,007609 | 7,549988 | 26,75719 | 9,76308 | 9,66449 | 2,760826 | 0,35809 | 9,455294 | 14,92685 | 2,049914 | 9,66245 | 18,60209 | 9,598732 | 9,504302 | 18,81215 | 18,35177 | 10,0681 | 7,65958 | 6,509112 | 9,850251 | 18,78462 | 19,42374 |
| 00071 Fatty acid degradation | Lipid metabolism | 4,797549 | 7,042224 | 0,658264 | 4,485118 | 12,01813 | 4,559346 | 5,23436 | 1,4712 | 0,159335 | 5,610135 | 9,601473 | 1,423849 | 3,172693 | 6,490566 | 2,261573 | 2,888897 | 5,308975 | 5,300359 | 3,066408 | 2,644145 | 1,375554 | 3,050067 | 6,601028 | 5,922236 |
| 00120 Primary bile acid biosynthesis | Lipid metabolism | 0,405866 | 8,179252 | 3,310843 | 0,901094 | 29,7981 | 28,15687 | 1,095915 | 1,702897 | 0,737487 | 0,897117 | 2,542878 | 0,451328 | 6,99417 | 5,193536 | 5,77782 | 6,672097 | 13,65674 | 5,778829 | 6,146566 | 4,869381 | 3,681282 | 7,270007 | 4,045971 | 2,501169 |
| 00121 Secondary bile acid biosynthesis | Lipid metabolism | 0,391267 | 8,179252 | 3,310843 | 0,883051 | 29,37283 | 28,15687 | 1,000654 | 1,702897 | 0,737487 | 0,809378 | 2,530071 | 0,376275 | 1,30016 | 1,121306 | 1,191441 | 1,263285 | 2,807207 | 1,14892 | 1,219706 | 1,323674 | 0,697811 | 1,392041 | 1,041895 | 0,60939 |
| 00130 Ubiquinone and other terpenoid-quinone biosynthesis | Metabolism of cofactors and vitamins | 4,386606 | 1,454273 | 0,362068 | 4,576824 | 9,206483 | 4,432147 | 5,251888 | 0,60559 | 0,067917 | 5,758668 | 5,955158 | 1,273815 | 4,510095 | 3,782445 | 3,210618 | 4,563415 | 5,126643 | 5,494265 | 4,33389 | 2,112919 | 1,97816 | 4,483316 | 4,841132 | 5,719024 |
| 00140 Steroid hormone biosynthesis | Lipid metabolism | 0,013157 | 0 | 0 | 0 | 0 | 0 | 0 | 0 | 0 | 0 | 0 | 0 | 0,444582 | 0 | 1,03363 | 0,398347 | 0 | 0 | 0,471204 | 0,747882 | 0,937487 | 0,32163 | 0 | 0,216613 |
| 00190 Oxidative phosphorylation | Energy metabolism | 6,358744 | 13,75126 | 1,055226 | 5,611483 | 17,35233 | 5,677802 | 6,947325 | 2,620627 | 0,298599 | 6,85413 | 14,18466 | 1,646988 | 9,95359 | 14,40708 | 9,056794 | 9,122744 | 13,33238 | 13,97313 | 9,678306 | 7,231392 | 6,59728 | 9,867326 | 13,59915 | 12,89257 |
| 00195 Photosynthesis | Energy metabolism | 26,78837 | 108,5439 | 7,885137 | 19,43345 | 81,14664 | 29,65064 | 25,65953 | 18,47621 | 2,075824 | 24,47025 | 83,8311 | 6,630953 | 37,25456 | 67,58225 | 32,81235 | 29,92025 | 43,7094 | 35,47893 | 37,02268 | 33,45582 | 27,52876 | 36,89095 | 43,37265 | 25,82806 |
| 00220 Arginine biosynthesis | Amino acid metabolism | 7,868995 | 24,76248 | 1,771049 | 6,376574 | 24,74331 | 7,023784 | 8,350263 | 4,723575 | 0,635636 | 8,161303 | 23,00384 | 2,20125 | 13,44095 | 16,73653 | 11,17349 | 11,38356 | 14,91249 | 13,04281 | 12,62554 | 10,401 | 9,078852 | 12,70529 | 13,51115 | 10,4274 |
| 00230 Purine metabolism | Nucleotide metabolism | 7,653117 | 18,08528 | 1,594921 | 6,717456 | 23,40475 | 8,246 | 8,265717 | 3,971408 | 0,487404 | 8,229088 | 19,61566 | 2,168178 | 9,795729 | 13,16274 | 7,646728 | 8,290911 | 13,28944 | 10,26534 | 9,962941 | 7,325771 | 5,857968 | 9,668053 | 11,63305 | 9,157397 |
| 00240 Pyrimidine metabolism | Nucleotide metabolism | 6,810448 | 17,45459 | 1,712731 | 5,937162 | 22,34391 | 8,781183 | 7,678977 | 3,676762 | 0,526438 | 7,555181 | 17,23968 | 1,861432 | 11,59364 | 15,29979 | 9,857557 | 9,689172 | 15,33022 | 13,28101 | 11,63436 | 8,809738 | 7,606891 | 11,61177 | 13,77871 | 11,49702 |
| 00250 Alanine, aspartate and glutamate metabolism | Amino acid metabolism | 7,746701 | 24,35857 | 2,026336 | 5,867287 | 25,9103 | 7,624962 | 8,023754 | 5,041735 | 0,642948 | 7,836049 | 23,4928 | 2,075844 | 15,02621 | 21,49989 | 13,39928 | 11,78442 | 20,35183 | 19,75963 | 13,73819 | 11,81105 | 10,21523 | 14,07534 | 19,75491 | 18,13806 |
| 00253 Tetracycline biosynthesis | Metabolism of terpenoids and polyketides | 0,660907 | 0 | 0 | 0,763312 | 0 | 0 | 2,706347 | 0 | 0 | 0,22066 | 0 | 0,03011 | 24,31843 | 4,635627 | 9,821207 | 29,27613 | 3,640455 | 4,52111 | 19,53803 | 9,39541 | 14,6234 | 23,12177 | 5,083533 | 6,083597 |
| 00260 Glycine, serine and threonine metabolism | Amino acid metabolism | 6,01326 | 11,96947 | 1,085283 | 5,14865 | 17,81866 | 4,966546 | 6,321485 | 2,747272 | 0,35165 | 6,67347 | 14,39673 | 1,622315 | 9,256688 | 12,13885 | 9,057896 | 8,461331 | 12,91709 | 12,83262 | 8,918633 | 6,990471 | 6,811698 | 9,063061 | 12,49652 | 12,29799 |
| 00261 Monobactam biosynthesis | Biosynthesis of other secondary metabolites | 8,377066 | 13,12283 | 0,822212 | 7,430585 | 22,3968 | 4,409821 | 9,271151 | 3,439447 | 0,457342 | 9,458842 | 18,19206 | 2,396796 | 11,42757 | 13,56283 | 10,86851 | 11,12698 | 14,65422 | 14,68385 | 11,51906 | 8,756216 | 9,357575 | 11,49431 | 13,38065 | 12,87163 |
| 00270 Cysteine and methionine metabolism | Amino acid metabolism | 7,540372 | 19,65549 | 2,387033 | 6,321419 | 21,57181 | 5,131814 | 7,54226 | 4,846719 | 1,248941 | 7,918986 | 19,1678 | 2,347984 | 8,475572 | 14,425 | 7,193346 | 7,461541 | 12,73147 | 10,54443 | 8,086872 | 7,599291 | 5,79425 | 7,718349 | 12,09986 | 10,31135 |
| 00280 Valine, leucine and isoleucine degradation | Amino acid metabolism | 3,660493 | 3,295411 | 2,083817 | 3,491649 | 6,546471 | 2,468493 | 3,980522 | 2,574462 | 1,40198 | 3,969066 | 4,918813 | 1,815903 | 2,193057 | 2,001967 | 1,374526 | 2,679165 | 1,320414 | 1,046628 | 2,071813 | 2,373648 | 1,441028 | 2,022527 | 2,046379 | 0,955488 |
| 00290 Valine, leucine and isoleucine biosynthesis | Amino acid metabolism | 15,92677 | 47,66936 | 7,355231 | 12,1046 | 39,11734 | 6,24905 | 15,49442 | 14,36457 | 4,263035 | 14,96296 | 44,87744 | 5,971356 | 21,14127 | 32,48885 | 16,13807 | 16,28747 | 24,44987 | 18,66714 | 19,25127 | 19,9437 | 14,23831 | 18,89594 | 24,97561 | 19,02554 |
| 00300 Lysine biosynthesis | Amino acid metabolism | 6,945306 | 17,91614 | 1,573334 | 6,263536 | 24,28082 | 10,60688 | 7,607653 | 3,853108 | 0,511432 | 7,933765 | 18,08214 | 2,083512 | 12,12843 | 21,19883 | 13,56366 | 11,11297 | 21,32403 | 22,63561 | 12,21281 | 9,968001 | 9,487464 | 12,03241 | 22,43972 | 22,68244 |
| 00310 Lysine degradation | Amino acid metabolism | 3,662728 | 4,947758 | 0,351243 | 3,328407 | 8,400807 | 1,466904 | 3,980153 | 1,247966 | 0,159997 | 4,238288 | 7,118946 | 0,971097 | 1,837597 | 2,353782 | 0,796866 | 2,038234 | 2,106765 | 1,275644 | 1,938338 | 1,595379 | 0,947684 | 1,802062 | 2,606395 | 1,040949 |
| 00311 Penicillin and cephalosporin biosynthesis | Biosynthesis of other secondary metabolites | 1,018852 | 0,584914 | 0 | 1,03694 | 2,278417 | 0,576397 | 1,086468 | 0,222541 | 0,007081 | 1,370291 | 2,273158 | 0,413821 | 1,241402 | 0,755354 | 0,487443 | 1,018668 | 2,42759 | 1,669556 | 1,748666 | 0,384995 | 0,356598 | 1,511367 | 1,314276 | 0,908435 |
| 00330 Arginine and proline metabolism | Amino acid metabolism | 3,89183 | 6,318865 | 0,639585 | 3,485897 | 10,98232 | 3,796865 | 4,170218 | 1,622717 | 0,19963 | 4,39396 | 8,891856 | 1,124583 | 3,550811 | 5,181905 | 3,118843 | 3,500876 | 5,308934 | 4,861093 | 3,326785 | 2,807745 | 2,198356 | 3,375097 | 5,417204 | 5,145563 |
| 00332 Carbapenem biosynthesis | Biosynthesis of other secondary metabolites | 20,66829 | 82,94816 | 6,853204 | 13,47649 | 73,53145 | 13,02886 | 19,00822 | 17,7769 | 2,366 | 17,21821 | 78,63369 | 5,507428 | 31,30685 | 65,58139 | 27,2355 | 24,15149 | 61,62644 | 50,0227 | 29,18237 | 28,84048 | 20,4072 | 27,71958 | 61,79867 | 57,9913 |
| 00333 Prodigiosin biosynthesis | Biosynthesis of other secondary metabolites | 9,979603 | 23,78814 | 0,929568 | 9,386708 | 29,76367 | 11,86539 | 11,61874 | 3,345985 | 0,460743 | 12,64321 | 18,60404 | 2,602242 | 10,29386 | 15,16193 | 6,499162 | 11,37853 | 9,728824 | 5,667698 | 11,34155 | 9,615079 | 6,473396 | 11,5529 | 12,93505 | 5,797213 |
| 00340 Histidine metabolism | Amino acid metabolism | 7,325105 | 32,8497 | 2,384724 | 5,100927 | 31,7725 | 5,078909 | 6,551427 | 6,371856 | 0,897352 | 6,747607 | 29,97606 | 2,193841 | 14,82065 | 22,19088 | 12,56182 | 11,39671 | 20,82991 | 16,5937 | 13,65576 | 11,90018 | 10,08971 | 13,36098 | 18,2915 | 16,36134 |
| 00350 Tyrosine metabolism | Amino acid metabolism | 2,514798 | 5,617115 | 0,511316 | 2,258425 | 8,963781 | 3,197518 | 2,727669 | 1,107562 | 0,222857 | 3,144604 | 6,162795 | 0,754277 | 2,158693 | 2,637973 | 1,195905 | 2,169351 | 2,014184 | 1,038327 | 2,027853 | 1,599998 | 0,905673 | 2,064817 | 1,512554 | 0,783451 |
| 00360 Phenylalanine metabolism | Amino acid metabolism | 2,684757 | 3,501155 | 0,25413 | 2,408892 | 7,610179 | 1,22916 | 2,787879 | 0,86965 | 0,122828 | 3,048403 | 4,993799 | 0,71684 | 2,944137 | 2,31205 | 1,744943 | 2,941748 | 1,965487 | 1,264199 | 2,89052 | 2,129795 | 1,449959 | 3,073469 | 2,330428 | 1,248899 |
| 00361 Chlorocyclohexane and chlorobenzene degradation | Xenobiotics biodegradation and metabolism | 1,743837 | 0,889762 | 0,431374 | 1,575424 | 4,740709 | 4,574664 | 1,47576 | 0,398348 | 0,029497 | 2,035304 | 3,66206 | 0,748073 | 0,470861 | 0,219876 | 0,281634 | 0,419996 | 0,1656 | 0,470571 | 0,476215 | 0,267125 | 0,073307 | 0,63972 | 0,401723 | 0,139148 |
| 00362 Benzoate degradation | Xenobiotics biodegradation and metabolism | 2,141227 | 2,54315 | 0,19246 | 1,896579 | 6,076179 | 1,195521 | 2,271537 | 0,695371 | 0,089753 | 2,550048 | 4,439924 | 0,61133 | 1,556311 | 1,687152 | 0,761395 | 1,54108 | 1,473092 | 0,620316 | 1,543095 | 1,151586 | 0,586165 | 1,549287 | 1,852211 | 0,561101 |
| 00364 Fluorobenzoate degradation | Xenobiotics biodegradation and metabolism | 2,5046 | 1,164907 | 0,057683 | 2,300087 | 5,446218 | 1,243381 | 2,260055 | 0,562984 | 0,056933 | 3,163226 | 4,855902 | 1,014718 | 0,366889 | 0,166587 | 0 | 0,274028 | 0 | 0 | 0,45803 | 0,302229 | 0,045972 | 0,544011 | 0,06897 | 0,082344 |
| 00380 Tryptophan metabolism | Amino acid metabolism | 3,206604 | 3,832636 | 0,296601 | 2,922971 | 8,150562 | 1,331487 | 3,523782 | 0,996704 | 0,113468 | 3,722062 | 6,555145 | 0,944988 | 2,380993 | 4,569833 | 1,840743 | 2,474565 | 4,244472 | 4,569429 | 2,084247 | 1,757623 | 1,056427 | 2,13632 | 5,014911 | 5,020905 |
| 00400 Phenylalanine, tyrosine and tryptophan biosynthesis | Amino acid metabolism | 5,576611 | 16,41662 | 1,237979 | 4,512091 | 21,54679 | 3,749369 | 5,830395 | 3,828013 | 0,497475 | 5,744585 | 17,36053 | 1,667423 | 11,86881 | 14,32474 | 9,304965 | 10,09695 | 14,57399 | 11,39184 | 11,19382 | 8,87564 | 7,439052 | 11,13575 | 12,87169 | 11,12646 |
| 00401 Novobiocin biosynthesis | Biosynthesis of other secondary metabolites | 4,727962 | 12,29512 | 0,930354 | 3,906328 | 15,73257 | 2,635861 | 4,647621 | 2,661927 | 0,392011 | 5,092131 | 13,81159 | 1,435743 | 6,878807 | 6,813609 | 3,658079 | 6,144321 | 5,373367 | 2,217672 | 6,432593 | 5,241602 | 3,317485 | 6,51937 | 5,005894 | 2,470116 |
| 00404 Staurosporine biosynthesis | Biosynthesis of other secondary metabolites | 0 | 0 | 0 | 0 | 0,069887 | 0 | 0 | 0 | 0 | 0 | 0 | 0 | NA | NA | NA | NA | NA | NA | NA | NA | NA | NA | NA | NA |
| 00405 Phenazine biosynthesis | Biosynthesis of other secondary metabolites | 0,380298 | 0 | 0,003162 | 0,421112 | 1,627074 | 0,202601 | 1,054837 | 0,466135 | 0 | 0,472739 | 0,634068 | 0,189485 | 1,927925 | 0,791105 | 1,192995 | 2,757172 | 0,751921 | 0,689754 | 0,991295 | 0,692786 | 0,455848 | 1,08891 | 0,573255 | 0,4976 |
| 00410 beta-Alanine metabolism | Metabolism of other amino acids | 5,866896 | 3,382194 | 3,053998 | 5,98211 | 7,784487 | 2,141162 | 6,426645 | 4,239987 | 2,350715 | 6,349447 | 5,424218 | 2,801902 | 4,58783 | 3,020195 | 4,467029 | 5,100933 | 3,838967 | 3,599376 | 4,9573 | 4,526409 | 4,460865 | 4,748938 | 3,115185 | 2,98267 |
| 00430 Taurine and hypotaurine metabolism | Metabolism of other amino acids | 3,303713 | 5,237463 | 0,816135 | 2,962811 | 7,648688 | 7,68073 | 3,856937 | 1,053415 | 0,098222 | 3,656242 | 4,88013 | 0,726033 | 6,265839 | 9,102328 | 5,140468 | 6,152114 | 8,408343 | 9,490815 | 6,36346 | 4,125967 | 3,104623 | 6,651992 | 9,706281 | 9,582743 |
| 00440 Phosphonate and phosphinate metabolism | Metabolism of other amino acids | 2,660815 | 6,461266 | 0,049829 | 2,742895 | 6,423615 | 1,806679 | 2,801526 | 0,774299 | 0,111848 | 3,397616 | 5,62921 | 0,778901 | 2,251774 | 3,856141 | 1,778053 | 2,954453 | 1,614125 | 1,072124 | 2,228674 | 1,291209 | 1,134317 | 2,516481 | 2,38321 | 1,145845 |
| 00450 Selenocompound metabolism | Metabolism of other amino acids | 8,428378 | 32,16175 | 2,282645 | 5,613957 | 28,11015 | 5,471099 | 7,692432 | 6,5799 | 0,76823 | 7,419072 | 29,65643 | 2,368875 | 11,04736 | 17,50865 | 8,033438 | 8,851108 | 13,26722 | 7,935347 | 10,79857 | 10,46418 | 8,195323 | 10,18161 | 11,07842 | 6,215541 |
| 00460 Cyanoamino acid metabolism | Metabolism of other amino acids | 9,768942 | 34,60704 | 3,688189 | 6,797582 | 33,70494 | 12,57373 | 8,547376 | 7,484861 | 0,894476 | 9,428949 | 36,02353 | 2,746577 | 17,83701 | 24,95966 | 15,36688 | 12,9445 | 22,01112 | 21,14304 | 15,45953 | 13,90717 | 12,21264 | 15,81075 | 21,9566 | 20,51878 |
| 00470 D-Amino acid metabolism | Metabolism of other amino acids | 5,962002 | 21,78256 | 1,755114 | 4,696961 | 21,93067 | 7,615087 | 5,762341 | 4,044748 | 0,490149 | 6,083343 | 20,71515 | 1,878905 | 8,293491 | 11,69236 | 5,153107 | 6,96162 | 10,0728 | 6,371633 | 8,162272 | 5,89965 | 4,024412 | 7,713198 | 8,447031 | 6,158659 |
| 00480 Glutathione metabolism | Metabolism of other amino acids | 5,439015 | 6,045799 | 0,91732 | 5,058724 | 14,01902 | 6,544802 | 5,866743 | 1,749073 | 0,223316 | 6,544133 | 10,65102 | 1,56359 | 3,410842 | 5,638871 | 2,74768 | 3,642283 | 5,183762 | 5,385415 | 3,014693 | 2,430453 | 1,364969 | 3,08726 | 5,691858 | 5,74824 |
| 00500 Starch and sucrose metabolism | Carbohydrate metabolism | 6,877425 | 24,91361 | 1,794683 | 5,551887 | 21,13166 | 7,260414 | 6,392287 | 4,654612 | 0,594934 | 6,955 | 22,36266 | 2,021609 | 8,746911 | 21,28002 | 7,829657 | 6,181527 | 17,61617 | 17,04673 | 8,037017 | 7,824947 | 5,16238 | 7,667676 | 17,72847 | 17,96352 |
| 00510 N-Glycan biosynthesis | Glycan biosynthesis and metabolism | 0,725213 | 0,070065 | 0 | 0,693148 | 2,112403 | 0,151261 | 1,03734 | 0,02874 | 0,025018 | 0,771518 | 0,247413 | 0,134385 | 4,060809 | 0,394617 | 3,04267 | 3,809617 | 0,533985 | 0,611772 | 2,951397 | 1,832505 | 1,618553 | 3,956047 | 0,900832 | 1,092495 |
| 00511 Other glycan degradation | Glycan biosynthesis and metabolism | 2,511881 | 10,01863 | 1,467498 | 2,66822 | 8,713704 | 8,461196 | 3,269587 | 2,285852 | 0,270584 | 2,516636 | 8,55199 | 0,703635 | 17,10098 | 26,8713 | 18,75437 | 15,16416 | 28,7399 | 34,51132 | 17,14231 | 11,48627 | 10,99509 | 17,42567 | 33,02844 | 38,4773 |
| 00513 Various types of N-glycan biosynthesis | Glycan biosynthesis and metabolism | 0 | 0 | 0 | 2,136845 | 0,091587 | 0 | 2,728185 | 0 | 0 | 0 | 0 | 0,092863 | 36,19585 | 133,2956 | 73,71495 | 35,7016 | 144,0184 | 208,2041 | 38,29734 | 27,36267 | 28,15309 | 38,62573 | 184,1301 | 241,5833 |
| 00520 Amino sugar and nucleotide sugar metabolism | Carbohydrate metabolism | 4,961687 | 16,78287 | 1,184404 | 4,315718 | 14,95126 | 6,796955 | 5,571409 | 2,813851 | 0,366111 | 5,409059 | 12,73143 | 1,288602 | 7,09819 | 15,42188 | 8,068093 | 6,256079 | 13,27511 | 14,2341 | 7,088333 | 6,083753 | 5,606703 | 6,885788 | 13,38724 | 13,74326 |
| 00521 Streptomycin biosynthesis | Biosynthesis of other secondary metabolites | 6,220838 | 20,27085 | 1,898301 | 5,297584 | 19,16925 | 7,092018 | 6,244796 | 4,644913 | 0,980883 | 6,560415 | 16,6133 | 1,286336 | 12,65966 | 34,21261 | 17,31001 | 11,16271 | 35,16777 | 37,08454 | 12,3749 | 11,80004 | 10,06347 | 11,77765 | 38,22178 | 42,14876 |
| 00523 Polyketide sugar unit biosynthesis | Metabolism of terpenoids and polyketides | 3,526324 | 6,267608 | 1,031384 | 3,831015 | 5,418825 | 1,55846 | 4,843074 | 3,876012 | 1,260695 | 4,001767 | 4,807787 | 0,981583 | 14,84093 | 50,1213 | 30,12994 | 15,23878 | 53,57314 | 65,95366 | 15,20664 | 17,01784 | 16,59442 | 14,20894 | 64,77945 | 77,26786 |
| 00524 Neomycin, kanamycin and gentamicin biosynthesis | Biosynthesis of other secondary metabolites | 10,25951 | 53,00757 | 2,190831 | 9,836516 | 17,73165 | 18,83409 | 10,33326 | 3,864981 | 0,198103 | 13,06791 | 24,88422 | 1,671959 | 17,19035 | 35,05667 | 7,486606 | 14,47044 | 16,23264 | 12,0483 | 17,89707 | 9,644122 | 6,682349 | 16,66067 | 15,90346 | 6,238634 |
| 00525 Acarbose and validamycin biosynthesis | Biosynthesis of other secondary metabolites | 4,752284 | 2,51585 | 0,138974 | 4,707775 | 5,444666 | 1,316243 | 6,229381 | 2,174583 | 0,93317 | 5,516051 | 6,47906 | 1,437974 | 15,22363 | 88,20849 | 45,82525 | 15,74091 | 90,61088 | 125,4064 | 14,37046 | 19,99651 | 19,81578 | 13,53215 | 114,8493 | 148,6188 |
| 00531 Glycosaminoglycan degradation | Glycan biosynthesis and metabolism | 1,549626 | 0,565085 | 0,497906 | 2,039392 | 3,607915 | 4,331915 | 2,487197 | 0,209858 | 0,009184 | 2,070343 | 1,883112 | 0,330735 | 12,03213 | 27,72912 | 16,01796 | 10,24202 | 31,98604 | 43,39731 | 11,58594 | 7,057319 | 6,8831 | 11,88075 | 39,1589 | 48,94763 |
| 00540 Lipopolysaccharide biosynthesis | Glycan biosynthesis and metabolism | 4,779187 | 1,444821 | 0,056434 | 4,990019 | 8,737511 | 1,802704 | 5,488402 | 0,6072 | 0,06449 | 5,993641 | 6,030471 | 1,278396 | 5,88705 | 3,517272 | 4,985092 | 6,125019 | 4,982754 | 5,768838 | 5,966272 | 3,096876 | 3,636792 | 6,248737 | 4,591965 | 5,502445 |
| 00541 O-Antigen nucleotide sugar biosynthesis | Glycan biosynthesis and metabolism | 3,659746 | 9,384419 | 0,750046 | 3,230865 | 8,312328 | 2,599342 | 3,933554 | 2,179441 | 0,500744 | 4,015899 | 8,129966 | 1,142058 | 5,520237 | 13,05734 | 8,123204 | 5,531144 | 12,34923 | 14,40067 | 5,45143 | 5,276467 | 5,510773 | 5,307595 | 13,84779 | 15,69528 |
| 00542 O-Antigen repeat unit biosynthesis | Glycan biosynthesis and metabolism | 5,068251 | 15,09363 | 0,311007 | 5,626604 | 12,37346 | 4,38284 | 4,900413 | 3,170843 | 0,478231 | 7,428711 | 12,79329 | 2,319144 | 1,565201 | 6,320962 | 0,775213 | 1,908348 | 5,238362 | 2,480691 | 2,74158 | 4,285536 | 1,622424 | 2,261662 | 3,239663 | 1,666488 |
| 00543 Exopolysaccharide biosynthesis | Glycan biosynthesis and metabolism | 1,62596 | 4,315557 | 0,041409 | 1,636207 | 3,832273 | 1,008801 | 1,753604 | 0,452953 | 0,061079 | 2,169451 | 3,30924 | 0,482953 | 1,646119 | 3,02389 | 0,665001 | 1,750941 | 1,330486 | 0,981985 | 1,659352 | 1,100008 | 0,603162 | 1,52733 | 1,858755 | 0,643731 |
| 00550 Peptidoglycan biosynthesis | Glycan biosynthesis and metabolism | 8,992362 | 35,26063 | 3,672991 | 6,700923 | 35,88802 | 15,44472 | 8,469531 | 7,409912 | 0,924473 | 8,769432 | 33,11934 | 2,849216 | 17,40293 | 25,72244 | 14,14142 | 13,06797 | 24,66033 | 20,90075 | 15,98577 | 13,56507 | 10,25766 | 16,01377 | 23,67547 | 19,89944 |
| 00552 Teichoic acid biosynthesis | Glycan biosynthesis and metabolism | 2,274853 | 7,756483 | 1,082307 | 1,967116 | 15,70128 | 7,830983 | 2,320877 | 1,658047 | 0,332666 | 2,75238 | 6,892674 | 0,734335 | 4,909376 | 8,195738 | 4,663944 | 4,232239 | 11,70729 | 9,338645 | 4,758943 | 3,4496 | 2,888574 | 4,82918 | 8,917976 | 9,320886 |
| 00561 Glycerolipid metabolism | Lipid metabolism | 3,34034 | 4,010176 | 0,931963 | 3,561729 | 11,66728 | 6,579426 | 4,074854 | 0,956405 | 0,141113 | 4,254013 | 5,460941 | 0,979301 | 3,616041 | 2,942044 | 1,974132 | 3,185797 | 4,125126 | 2,079672 | 3,762012 | 2,105408 | 1,010355 | 3,833245 | 3,105229 | 1,340208 |
| 00562 Inositol phosphate metabolism | Carbohydrate metabolism | 4,563624 | 13,01101 | 1,400336 | 3,408891 | 19,13108 | 6,078159 | 4,481083 | 2,664676 | 0,437391 | 4,59507 | 13,59561 | 1,059754 | 6,620591 | 13,10705 | 7,139152 | 5,263482 | 13,94103 | 13,35593 | 5,931123 | 5,758059 | 5,090463 | 5,894509 | 12,98709 | 12,82961 |
| 00564 Glycerophospholipid metabolism | Lipid metabolism | 5,123225 | 5,504101 | 0,66164 | 4,992455 | 13,07941 | 5,872336 | 5,665552 | 1,421917 | 0,181893 | 6,301129 | 8,4657 | 1,346805 | 6,073485 | 7,566327 | 5,190603 | 5,795091 | 8,923872 | 8,038243 | 5,482054 | 4,242661 | 3,518986 | 5,702253 | 8,750199 | 8,241166 |
| 00565 Ether lipid metabolism | Lipid metabolism | 4,095991 | 1,201087 | 0,046283 | 3,924004 | 7,702968 | 1,476564 | 3,890768 | 0,458783 | 0,060921 | 4,904558 | 4,965063 | 1,077131 | 0,646168 | 0,301246 | 0,185408 | 1,070273 | 0,283926 | 0,264509 | 0,261282 | 0,143433 | 0,052291 | 0,235798 | 0,010699 | 0,07608 |
| 00572 Arabinogalactan biosynthesis - Mycobacterium | Glycan biosynthesis and metabolism | 5,068251 | 15,09363 | 0,311007 | 5,626604 | 12,37346 | 4,38284 | 4,900413 | 3,170843 | 0,478231 | 7,428711 | 12,79329 | 2,319144 | 1,565201 | 6,320962 | 0,775213 | 1,908348 | 5,238362 | 2,480691 | 2,74158 | 4,285536 | 1,622424 | 2,261662 | 3,239663 | 1,666488 |
| 00590 Arachidonic acid metabolism | Lipid metabolism | 4,085875 | 1,201087 | 0,046283 | 3,948092 | 7,660687 | 1,476564 | 3,885675 | 0,458783 | 0,060921 | 4,906772 | 4,893321 | 1,077131 | 0,423893 | 0,157581 | 0 | 0,495428 | 0,324287 | 0,107783 | 0,391923 | 0,215149 | 0,078437 | 0,345219 | 0,00831 | 0 |
| 00591 Linoleic acid metabolism | Lipid metabolism | 8,151468 | 2,402175 | 0,092566 | 7,848008 | 14,78184 | 2,953129 | 7,679543 | 0,917565 | 0,121842 | 9,788222 | 9,747184 | 2,154262 | 0,801894 | 0,315161 | 0 | 0,899779 | 0,608272 | 0,215566 | 0,783846 | 0,430298 | 0,156874 | 0,690438 | 0,01662 | 0 |
| 00592 alpha-Linolenic acid metabolism | Lipid metabolism | 13,12195 | 4,134147 | 0,184222 | 12,90306 | 25,29451 | 5,852241 | 14,36876 | 1,661837 | 0,185236 | 16,48138 | 18,15888 | 3,926906 | 2,391922 | 2,042392 | 0,362111 | 3,29493 | 1,543323 | 0,776242 | 3,009937 | 1,508071 | 0,459959 | 2,912937 | 4,690306 | 0,793373 |
| 00600 Sphingolipid metabolism | Lipid metabolism | 3,010472 | 10,85967 | 2,707076 | 3,368333 | 11,28583 | 9,701459 | 3,626095 | 2,419323 | 0,298128 | 3,063297 | 10,71374 | 0,92304 | 20,29719 | 25,26319 | 19,01575 | 16,51286 | 28,54706 | 31,37263 | 19,90768 | 12,58316 | 11,42196 | 19,99114 | 30,39747 | 34,48797 |
| 00603 Glycosphingolipid biosynthesis - globo and isoglobo series | Glycan biosynthesis and metabolism | 2,270339 | 1,513077 | 2,895738 | 4,117727 | 7,544912 | 2,582538 | 4,019576 | 0,45367 | 0,09854 | 3,006986 | 3,11585 | 0,622372 | 23,16517 | 47,47378 | 30,29934 | 19,51008 | 56,52062 | 73,39448 | 24,34466 | 13,61175 | 11,71643 | 24,50328 | 65,82569 | 83,77813 |
| 00604 Glycosphingolipid biosynthesis - ganglio series | Glycan biosynthesis and metabolism | 0 | 0 | 0 | 2,136845 | 0,091587 | 0 | 2,728185 | 0 | 0 | 0 | 0 | 0,092863 | 36,19585 | 133,2956 | 73,71495 | 35,7016 | 144,0184 | 208,2041 | 38,29734 | 27,36267 | 28,15309 | 38,62573 | 184,1301 | 241,5833 |
| 00620 Pyruvate metabolism | Carbohydrate metabolism | 4,827345 | 8,677976 | 0,79428 | 4,581011 | 14,32046 | 5,857603 | 5,633137 | 1,806085 | 0,25164 | 5,671615 | 9,39459 | 1,226974 | 4,60161 | 6,812163 | 3,989198 | 4,674073 | 6,072442 | 5,272186 | 4,614871 | 3,676893 | 2,911358 | 4,638543 | 5,73619 | 4,624902 |
| 00621 Dioxin degradation | Xenobiotics biodegradation and metabolism | 1,335174 | 0,500173 | 0 | 1,355275 | 4,767989 | 1,254861 | 2,053626 | 0,141156 | 0,082485 | 1,747471 | 0,560956 | 0,078533 | 1,911666 | 0,292397 | 0,950673 | 2,267794 | 1,483131 | 0,75499 | 1,702442 | 0,644971 | 0,783544 | 2,298719 | 0,703746 | 0,631819 |
| 00622 Xylene degradation | Xenobiotics biodegradation and metabolism | 0,814871 | 0,52768 | 0,008057 | 0,829048 | 4,643393 | 1,229145 | 1,246872 | 0,116897 | 0,070342 | 1,082421 | 0,303185 | 0,064799 | 1,228388 | 0,266514 | 0,620203 | 1,422168 | 1,595123 | 0,536163 | 1,101767 | 0,484745 | 0,509078 | 1,472692 | 0,450594 | 0,401188 |
| 00623 Toluene degradation | Xenobiotics biodegradation and metabolism | 3,056734 | 2,022456 | 0,098397 | 2,741689 | 11,38527 | 2,817809 | 2,616768 | 0,769408 | 0,109099 | 3,622408 | 6,168042 | 1,322398 | 0,337289 | 0,300614 | 0,041651 | 0,233007 | 1,063338 | 0,102871 | 0,329374 | 0,26721 | 0,067902 | 0,423832 | 0,085904 | 0,092597 |
| 00624 Polycyclic aromatic hydrocarbon degradation | Xenobiotics biodegradation and metabolism | 0,080698 | 0 | 0 | 0,099145 | 0,732238 | 0,010997 | 0,168059 | 0 | 0 | 0,192079 | 0,135472 | 0,003303 | 0,182034 | 0 | 0 | 0,159524 | 0 | 0 | 0,189906 | 0,125959 | 0,015919 | 0,269648 | 0,004478 | 0,005682 |
| 00625 Chloroalkane and chloroalkene degradation | Xenobiotics biodegradation and metabolism | 1,369391 | 5,113714 | 0,786514 | 1,299642 | 6,12778 | 7,396724 | 1,827562 | 0,715137 | 0,05844 | 1,647204 | 3,181354 | 0,431875 | 0,796325 | 3,620353 | 0,90472 | 0,927826 | 1,564781 | 1,385869 | 0,994126 | 1,070309 | 0,326927 | 0,927158 | 2,672495 | 0,5206 |
| 00626 Naphthalene degradation | Xenobiotics biodegradation and metabolism | 1,54331 | 5,518409 | 0,761656 | 1,482191 | 6,809129 | 7,101437 | 2,080317 | 0,47896 | 0,045065 | 1,868796 | 3,54216 | 0,440752 | 0,650576 | 3,260103 | 0,709481 | 0,7116 | 1,490797 | 1,400816 | 0,891717 | 0,762018 | 0,268096 | 0,843707 | 1,476747 | 0,330163 |
| 00627 Aminobenzoate degradation | Xenobiotics biodegradation and metabolism | 1,242914 | 1,487238 | 0,271423 | 1,279015 | 5,596095 | 3,365697 | 1,496473 | 0,405446 | 0,082234 | 1,680823 | 2,289865 | 0,43743 | 0,663055 | 0,85375 | 0,406202 | 0,877212 | 1,626066 | 0,835176 | 0,778523 | 0,561382 | 0,201995 | 0,689189 | 1,008697 | 0,425268 |
| 00630 Glyoxylate and dicarboxylate metabolism | Carbohydrate metabolism | 5,95095 | 11,49504 | 1,19449 | 5,325682 | 14,3765 | 4,251784 | 6,356859 | 2,557526 | 0,437753 | 6,530772 | 12,90991 | 1,571403 | 7,567343 | 9,902542 | 6,93937 | 6,887717 | 9,425919 | 9,627849 | 7,161655 | 5,666211 | 5,324212 | 7,248077 | 9,588559 | 8,494334 |
| 00633 Nitrotoluene degradation | Xenobiotics biodegradation and metabolism | 0,402261 | 0,22508 | 0 | 0,405242 | 1,881749 | 0,359039 | 0,541911 | 0,135665 | 0,006036 | 0,548906 | 1,391242 | 0,263022 | 0,272263 | 0,222041 | 0,060685 | 0,199678 | 0,144393 | 0,064016 | 0,231097 | 0,206713 | 0,032776 | 0,252179 | 0,114134 | 0,023016 |
| 00640 Propanoate metabolism | Carbohydrate metabolism | 4,037456 | 9,240256 | 0,730766 | 3,715459 | 11,73534 | 4,65687 | 4,816321 | 1,745724 | 0,245811 | 4,565979 | 8,969829 | 1,052888 | 3,366247 | 5,914659 | 2,321473 | 3,325605 | 4,329992 | 3,45477 | 3,446946 | 2,686653 | 1,717459 | 3,392275 | 4,139345 | 2,957292 |
| 00642 Ethylbenzene degradation | Xenobiotics biodegradation and metabolism | 9,089634 | 2,933059 | 0,137939 | 9,071666 | 18,35634 | 4,375677 | 10,61034 | 1,203054 | 0,124315 | 11,76947 | 13,31335 | 2,849775 | 2,285193 | 1,884811 | 0,362111 | 3,040095 | 1,239187 | 0,668459 | 2,858803 | 1,484565 | 0,381522 | 2,930977 | 4,681995 | 0,793373 |
| 00643 Styrene degradation | Xenobiotics biodegradation and metabolism | 0,316959 | 0,077335 | 0 | 0,331008 | 1,584069 | 0,20213 | 0,615296 | 0,031754 | 0,004229 | 0,572687 | 0,653466 | 0,150179 | 0,62963 | 0,507885 | 0,26648 | 0,643235 | 0,428107 | 0,250318 | 0,619126 | 0,329761 | 0,060693 | 0,600729 | 1,640009 | 0,249991 |
| 00650 Butanoate metabolism | Carbohydrate metabolism | 3,38204 | 4,806992 | 0,434893 | 3,206453 | 8,464583 | 2,656524 | 3,751957 | 1,138011 | 0,13467 | 3,964842 | 7,015655 | 0,986304 | 2,958444 | 4,752821 | 2,640595 | 2,855023 | 4,213356 | 4,597767 | 2,871134 | 2,335326 | 1,838789 | 2,936212 | 5,444667 | 4,57715 |
| 00660 C5-Branched dibasic acid metabolism | Carbohydrate metabolism | 9,081444 | 28,18264 | 2,080815 | 7,051358 | 24,81447 | 3,872554 | 9,094211 | 6,242895 | 0,919237 | 9,044357 | 28,08321 | 2,500363 | 10,70317 | 17,71849 | 7,663064 | 7,961205 | 13,6781 | 11,47807 | 9,810908 | 9,069402 | 6,201714 | 9,553077 | 14,79202 | 11,72461 |
| 00670 One carbon pool by folate | Metabolism of cofactors and vitamins | 9,540367 | 30,71093 | 3,220464 | 7,266962 | 33,50249 | 11,20778 | 9,929535 | 6,945605 | 0,863709 | 9,776143 | 31,35739 | 2,656197 | 21,94177 | 25,85751 | 22,84278 | 18,00467 | 27,7196 | 26,80242 | 21,19867 | 17,72849 | 18,00092 | 21,55474 | 25,2139 | 22,35124 |
| 00680 Methane metabolism | Energy metabolism | 5,347019 | 9,723563 | 0,975959 | 4,808775 | 15,24434 | 5,260377 | 5,954359 | 2,264549 | 0,320234 | 5,999433 | 11,03222 | 1,268626 | 9,649951 | 13,55309 | 10,09518 | 8,46134 | 14,76496 | 15,9243 | 9,091679 | 6,886558 | 7,123671 | 9,48702 | 14,87505 | 15,60592 |
| 00710 Carbon fixation in photosynthetic organisms | Energy metabolism | 7,67244 | 17,7794 | 1,705271 | 6,830675 | 21,6512 | 6,719854 | 8,774706 | 4,128565 | 0,606149 | 8,389461 | 18,94642 | 1,951144 | 14,27712 | 18,46757 | 13,34197 | 12,56614 | 17,7557 | 17,79399 | 14,4367 | 10,40674 | 10,09711 | 14,65821 | 18,14935 | 17,80668 |
| 00720 Carbon fixation pathways in prokaryotes | Energy metabolism | 5,436613 | 9,374656 | 0,739214 | 5,334496 | 14,07063 | 5,528457 | 6,867457 | 1,922045 | 0,277975 | 6,275934 | 9,130317 | 1,264302 | 6,766072 | 10,22002 | 6,902668 | 6,570307 | 9,785832 | 10,61937 | 6,663736 | 5,101662 | 4,663755 | 6,906657 | 10,70151 | 10,6649 |
| 00730 Thiamine metabolism | Metabolism of cofactors and vitamins | 9,805343 | 27,72793 | 2,943641 | 8,196595 | 31,36542 | 13,2731 | 9,896922 | 6,122606 | 0,803101 | 10,80439 | 28,89812 | 2,680804 | 15,63658 | 16,56712 | 11,25762 | 12,74382 | 17,73202 | 10,52239 | 15,36032 | 11,3112 | 9,435701 | 15,17146 | 13,81453 | 7,908012 |
| 00740 Riboflavin metabolism | Metabolism of cofactors and vitamins | 4,880483 | 5,329111 | 1,175721 | 5,228499 | 12,74443 | 6,631403 | 5,790385 | 2,294137 | 0,206138 | 6,179601 | 9,286788 | 1,819278 | 7,33657 | 6,489825 | 6,382574 | 6,557143 | 8,200707 | 7,265859 | 6,869005 | 4,895282 | 4,614286 | 7,339732 | 7,769958 | 6,120062 |
| 00750 Vitamin B6 metabolism | Metabolism of cofactors and vitamins | 9,086948 | 27,37297 | 2,450844 | 7,060298 | 28,09322 | 6,805832 | 8,997705 | 6,133833 | 0,732788 | 9,17555 | 27,51017 | 2,456536 | 16,99922 | 15,53769 | 9,907424 | 13,03546 | 17,32044 | 9,032523 | 15,95572 | 10,77005 | 8,351239 | 15,53949 | 10,95341 | 6,688812 |
| 00760 Nicotinate and nicotinamide metabolism | Metabolism of cofactors and vitamins | 4,811999 | 13,34783 | 1,686216 | 3,870367 | 17,45113 | 7,543904 | 5,099954 | 3,056105 | 0,380464 | 4,932643 | 14,54872 | 1,417686 | 6,815241 | 8,555803 | 4,473751 | 5,44223 | 8,757656 | 6,071146 | 6,377003 | 4,834939 | 3,289981 | 6,190932 | 7,637379 | 5,82252 |
| 00770 Pantothenate and CoA biosynthesis | Metabolism of cofactors and vitamins | 13,09328 | 28,84479 | 7,034008 | 11,55083 | 28,15229 | 9,461738 | 13,21801 | 11,24544 | 4,241936 | 13,16575 | 27,12079 | 5,225848 | 14,99721 | 17,36015 | 11,51624 | 12,88313 | 15,14267 | 10,10642 | 14,73618 | 13,50758 | 10,6241 | 14,29182 | 12,64815 | 8,186349 |
| 00780 Biotin metabolism | Metabolism of cofactors and vitamins | 6,66651 | 7,855545 | 0,426764 | 6,823522 | 18,32037 | 6,493935 | 7,812329 | 1,84549 | 0,214837 | 8,409925 | 11,46782 | 1,972327 | 10,18985 | 10,64246 | 7,572187 | 10,01272 | 12,44235 | 10,4357 | 10,01656 | 7,036704 | 5,538763 | 10,40583 | 11,96852 | 11,13324 |
| 00785 Lipoic acid metabolism | Metabolism of cofactors and vitamins | 7,471136 | 6,948216 | 1,783376 | 8,111507 | 22,62055 | 16,63297 | 9,843596 | 1,83521 | 0,317957 | 10,04033 | 12,07568 | 2,143768 | 17,16381 | 26,35983 | 19,20414 | 16,54338 | 31,75995 | 39,84337 | 17,25983 | 8,862415 | 10,05704 | 17,62144 | 33,93463 | 41,95613 |
| 00790 Folate biosynthesis | Metabolism of cofactors and vitamins | 4,459096 | 8,638621 | 0,94888 | 4,157153 | 12,07743 | 3,27076 | 4,48035 | 2,351693 | 0,226813 | 5,196053 | 11,29144 | 1,475256 | 7,861872 | 6,850191 | 6,34459 | 7,115363 | 6,531222 | 5,872487 | 7,399186 | 5,456492 | 5,297421 | 7,748854 | 7,036427 | 4,627984 |
| 00791 Atrazine degradation | Xenobiotics biodegradation and metabolism | 4,68793 | 1,078446 | 0,052072 | 4,959879 | 7,192794 | 1,480259 | 5,555132 | 0,474212 | 0,091708 | 6,135481 | 4,334421 | 1,007353 | 1,482736 | 0,455571 | 0,216913 | 2,133446 | 0,378742 | 0,345979 | 1,400698 | 0,569173 | 0,148199 | 1,488241 | 0,151006 | 0,184413 |
| 00830 Retinol metabolism | Metabolism of cofactors and vitamins | 1,377702 | 0,578435 | 0,851654 | 1,481127 | 6,55598 | 7,657641 | 1,833611 | 0,103704 | 0,046272 | 1,876179 | 0,850225 | 0,303776 | 0,955908 | 0,995116 | 1,709338 | 1,200117 | 1,277711 | 0,870189 | 1,063098 | 1,426204 | 1,839269 | 0,858707 | 0,71023 | 0,325028 |
| 00860 Porphyrin metabolism | Metabolism of cofactors and vitamins | 3,022567 | 3,147653 | 0,222983 | 2,914136 | 6,962704 | 1,681063 | 3,626624 | 0,954211 | 0,09113 | 3,685557 | 5,259974 | 0,823285 | 4,346521 | 3,283442 | 2,936738 | 4,75565 | 3,425141 | 2,461912 | 4,257186 | 2,783314 | 2,392522 | 4,497027 | 4,254679 | 2,210183 |
| 00900 Terpenoid backbone biosynthesis | Metabolism of terpenoids and polyketides | 8,779191 | 35,79014 | 3,180819 | 6,273427 | 30,94763 | 10,25142 | 8,128843 | 7,167067 | 0,820944 | 8,308127 | 33,30266 | 2,567434 | 14,51733 | 18,72809 | 8,377309 | 11,38376 | 14,35763 | 6,040139 | 14,23364 | 10,84822 | 7,052079 | 13,80998 | 12,25755 | 5,179945 |
| 00903 Limonene and pinene degradation | Metabolism of terpenoids and polyketides | 0,679868 | 11,25507 | 0,176032 | 0,699731 | 6,701016 | 1,677243 | 1,610584 | 2,234877 | 0,268946 | 1,033446 | 3,626784 | 0,63566 | 0,883363 | 3,975792 | 0,847065 | 0,837817 | 3,023788 | 1,447152 | 1,682277 | 2,281877 | 0,787481 | 1,256431 | 6,473341 | 1,431804 |
| 00906 Carotenoid biosynthesis | Metabolism of terpenoids and polyketides | 0,089928 | 2,258732 | 0,110439 | 0,029407 | 15,70458 | 4,899803 | 0,068673 | 0,316714 | 0,300021 | 0,12253 | 0,100667 | 0,035553 | 0,259941 | 1,247732 | 0,347619 | 0,172615 | 6,637908 | 0,933763 | 0,220354 | 0,554364 | 0,21561 | 0,300252 | 0,471501 | 0,23941 |
| 00908 Zeatin biosynthesis | Metabolism of terpenoids and polyketides | 25,2462 | 80,334 | 8,49748 | 18,71739 | 83,51481 | 23,96528 | 24,03891 | 16,11108 | 2,071004 | 23,85717 | 81,10307 | 7,114499 | 60,77974 | 43,68513 | 33,05127 | 49,00778 | 51,96416 | 23,53029 | 57,28869 | 32,8739 | 28,15135 | 57,04363 | 39,17559 | 21,73184 |
| 00910 Nitrogen metabolism | Energy metabolism | 4,316583 | 11,15615 | 0,958611 | 3,533403 | 11,036 | 3,307024 | 4,707109 | 2,311743 | 0,261858 | 4,553227 | 11,44905 | 1,059297 | 7,817527 | 9,761522 | 6,345561 | 6,487539 | 9,267869 | 8,947266 | 7,209367 | 5,593811 | 5,073656 | 7,152222 | 8,886626 | 7,190065 |
| 00920 Sulfur metabolism | Energy metabolism | 4,620391 | 6,930883 | 0,406938 | 4,327245 | 10,69327 | 2,4591 | 5,348254 | 1,416972 | 0,162074 | 5,431727 | 8,466942 | 1,124085 | 4,37739 | 5,289732 | 2,776164 | 4,168942 | 4,847285 | 3,943042 | 4,519105 | 2,736393 | 2,303949 | 4,311375 | 3,931302 | 3,332129 |
| 00930 Caprolactam degradation | Xenobiotics biodegradation and metabolism | 2,120018 | 0,687495 | 0,030459 | 2,108108 | 4,615438 | 1,01814 | 2,539139 | 0,333401 | 0,029294 | 2,584899 | 3,219924 | 0,756241 | 0,763205 | 0,479936 | 0,181091 | 1,029723 | 0,436089 | 0,411405 | 0,82926 | 0,552588 | 0,190682 | 0,782469 | 1,054499 | 0,366566 |
| 00940 Phenylpropanoid biosynthesis | Biosynthesis of other secondary metabolites | 6,435379 | 2,227496 | 0,098785 | 6,579435 | 13,13269 | 2,358641 | 6,672426 | 0,891737 | 0,106185 | 7,672674 | 9,442147 | 1,950475 | 1,563682 | 0,439159 | 0,192466 | 2,141127 | 0,66904 | 0,315228 | 1,52029 | 0,714943 | 0,226114 | 1,41081 | 0,139293 | 0,21978 |
| 00944 Flavone and flavonol biosynthesis | Biosynthesis of other secondary metabolites | 0 | 0,830961 | 2,46556 | 0 | 3,364613 | 19,09967 | 0 | 0 | 0 | 0 | 0,547155 | 0,113271 | 2,711067 | 0,730396 | 2,302052 | 1,36697 | 0,79891 | 2,913807 | 1,650027 | 1,88535 | 0,228386 | 2,019417 | 0,290281 | 0,122873 |
| 00950 Isoquinoline alkaloid biosynthesis | Biosynthesis of other secondary metabolites | 2,668358 | 3,687621 | 0,103169 | 2,794484 | 5,676433 | 1,282815 | 3,253608 | 0,897817 | 0,14439 | 3,67333 | 4,095331 | 0,739465 | 2,126005 | 1,584796 | 1,316469 | 2,718822 | 1,137159 | 0,753544 | 2,190373 | 1,960989 | 1,042106 | 2,377905 | 1,716397 | 0,8101 |
| 00960 Tropane, piperidine and pyridine alkaloid biosynthesis | Biosynthesis of other secondary metabolites | 4,467811 | 12,04632 | 0,92504 | 3,69645 | 14,45546 | 2,271921 | 4,407745 | 2,559425 | 0,382212 | 4,77091 | 12,5208 | 1,192898 | 6,793635 | 6,653917 | 3,527996 | 6,176655 | 5,270288 | 2,160012 | 6,382421 | 5,070846 | 3,176406 | 6,434654 | 4,527868 | 2,402088 |
| 00965 Betalain biosynthesis | Biosynthesis of other secondary metabolites | 3,816204 | 2,153597 | 0,067621 | 3,965011 | 8,05474 | 2,46736 | 3,524863 | 0,854697 | 0,067649 | 5,151164 | 8,312113 | 1,734993 | 0,644747 | 0,412602 | 0 | 0,439689 | 0,262964 | 0,088999 | 0,605113 | 0,377945 | 0,095519 | 0,715932 | 0,042495 | 0 |
| 00966 Glucosinolate biosynthesis | Biosynthesis of other secondary metabolites | 33,34932 | 57,00489 | 30,54043 | 29,58099 | 41,59771 | 10,21505 | 29,84737 | 39,24165 | 21,52447 | 30,5855 | 49,29275 | 20,33984 | 27,40859 | 60,54283 | 30,50683 | 25,06705 | 45,5838 | 54,6998 | 23,03693 | 35,61415 | 25,26199 | 22,07599 | 58,8209 | 65,87884 |
| 00970 Aminoacyl-tRNA biosynthesis | Translation | 13,83841 | 49,07306 | 5,106691 | 9,887476 | 51,36671 | 20,07431 | 13,99045 | 10,65068 | 1,370356 | 12,75146 | 45,9683 | 3,732139 | 27,32243 | 42,68412 | 25,9187 | 21,33632 | 41,11875 | 38,16108 | 26,7996 | 22,33138 | 19,39537 | 26,05932 | 38,14752 | 35,67853 |
| 00980 Metabolism of xenobiotics by cytochrome P450 | Xenobiotics biodegradation and metabolism | 5,472073 | 2,209634 | 0,710548 | 5,450481 | 12,84947 | 7,519839 | 5,67967 | 0,78453 | 0,108324 | 6,889331 | 7,651502 | 1,436917 | 1,256098 | 0,794228 | 0,537706 | 1,465342 | 1,108426 | 0,963519 | 1,268706 | 0,750005 | 0,112255 | 1,373318 | 0,308283 | 0,149522 |
| 00981 Insect hormone biosynthesis | Metabolism of terpenoids and polyketides | 0,679868 | 11,25507 | 0,176032 | 0,699731 | 6,701016 | 1,677243 | 1,610584 | 2,234877 | 0,268946 | 1,033446 | 3,626784 | 0,63566 | 1,071872 | 5,892009 | 1,299143 | 0,924344 | 3,847244 | 0,929413 | 2,433842 | 3,895564 | 1,003477 | 1,752697 | 5,877464 | 1,037265 |
| 00982 Drug metabolism - cytochrome P450 | Xenobiotics biodegradation and metabolism | 5,472073 | 2,209634 | 0,710548 | 5,450481 | 12,84947 | 7,519839 | 5,67967 | 0,78453 | 0,108324 | 6,889331 | 7,651502 | 1,436917 | 1,004878 | 0,635382 | 0,430165 | 1,172274 | 0,886741 | 0,770815 | 1,054631 | 0,649284 | 0,093107 | 1,117933 | 0,250116 | 0,119617 |
| 00983 Drug metabolism - other enzymes | Xenobiotics biodegradation and metabolism | 9,301725 | 15,29734 | 1,692845 | 8,803366 | 23,52645 | 10,10007 | 10,68806 | 3,333685 | 0,534669 | 10,68501 | 19,13369 | 2,301168 | 15,68796 | 17,13898 | 12,86382 | 13,38638 | 18,91729 | 18,05451 | 15,72751 | 10,07564 | 9,853389 | 15,68295 | 17,88937 | 15,87567 |
| 00996 Biosynthesis of various alkaloids | Biosynthesis of other secondary metabolites | 0 | 0 | 0 | 0 | 0 | 0 | 0,01742 | 0 | 0 | 0 | 0 | 0 | NA | NA | NA | NA | NA | NA | NA | NA | NA | NA | NA | NA |
| 00997 Biosynthesis of various other secondary metabolites | Biosynthesis of other secondary metabolites | 1,46749 | 0,92756 | 0,025399 | 1,476859 | 3,158358 | 0,938304 | 1,2773 | 0,412685 | 0,038446 | 2,020543 | 3,551852 | 0,624159 | 0,431405 | 0,3742 | 0,1336 | 0,635028 | 0,10523 | 0,075286 | 0,174318 | 0,143341 | 0 | 0,222579 | 0 | 0,071108 |
| 00998 Biosynthesis of various antibiotics | Biosynthesis of other secondary metabolites | 156,456 | 229,7087 | 153,0169 | 168,7632 | 280,8516 | 347,8911 | 167,7697 | 117,5809 | 232,6108 | 182,5072 | 315,7476 | 279,3349 | 146,6849 | 108,8627 | 109,0656 | 126,6909 | 74,69047 | 124,3221 | 156,1425 | 105,3383 | 143,5177 | 157,4963 | 110,6786 | 104,5274 |
| 00999 Biosynthesis of various plant secondary metabolites | Biosynthesis of other secondary metabolites | 14,13333 | 64,48674 | 6,166597 | 9,245979 | 58,1813 | 17,41358 | 11,49649 | 13,40633 | 1,62089 | 12,13427 | 61,74385 | 4,152611 | 42,45675 | 54,82715 | 34,41712 | 28,4748 | 53,9533 | 50,19354 | 35,44791 | 32,55086 | 28,14822 | 36,49381 | 47,4677 | 46,02159 |
| 01040 Biosynthesis of unsaturated fatty acids | Lipid metabolism | 1,571533 | 3,626133 | 0,102598 | 1,537836 | 5,587405 | 1,453315 | 1,720165 | 0,934656 | 0,090736 | 2,232221 | 4,90417 | 0,855478 | 0,627549 | 1,466971 | 0,246838 | 1,252221 | 0,958326 | 0,457935 | 0,793517 | 1,097856 | 0,252079 | 0,603436 | 0,342898 | 0,098006 |
| 01051 Biosynthesis of ansamycins | Metabolism of terpenoids and polyketides | 27,30682 | 94,68988 | 8,10119 | 17,68411 | 93,17586 | 11,10369 | 23,21295 | 21,2904 | 3,003994 | 23,27706 | 99,08714 | 7,060171 | 38,84058 | 44,02381 | 22,85981 | 32,81421 | 34,72663 | 8,829853 | 31,03157 | 34,49913 | 17,7204 | 34,89416 | 25,09686 | 11,6545 |
| 01053 Biosynthesis of siderophore group nonribosomal peptides | Metabolism of terpenoids and polyketides | 0,780002 | 0,130676 | 0,006098 | 0,901389 | 1,717271 | 0,293732 | 1,362097 | 0,035673 | 0,006799 | 1,301273 | 0,931871 | 0,162614 | 0,909409 | 0,276788 | 0,28342 | 1,086009 | 0,837074 | 0,484002 | 1,060614 | 0,148579 | 0,115935 | 1,014348 | 0,469991 | 0,316925 |
| 01054 Nonribosomal peptide structures | Metabolism of terpenoids and polyketides | 3,831593 | 2,459947 | 0,126316 | 3,495026 | 12,5094 | 3,328873 | 3,852626 | 0,9105 | 0,078843 | 4,040238 | 11,21349 | 2,255858 | 0,376686 | 0,409523 | 0 | 0,272661 | 0 | 0 | 0,416053 | 0,26896 | 0 | 0,748678 | 0 | 0 |
| 01055 Biosynthesis of vancomycin group antibiotics | Metabolism of terpenoids and polyketides | 5,231874 | 2,579136 | 0,220547 | 4,30344 | 5,064698 | 1,204767 | 6,810124 | 1,268941 | 0,883126 | 5,155797 | 6,407087 | 1,41683 | 14,92016 | 56,63525 | 35,1278 | 15,6254 | 53,95693 | 77,26895 | 13,12926 | 16,57945 | 17,35307 | 14,89006 | 71,41184 | 91,26547 |
| 02010 ABC transporters | Membrane transport | 4,994561 | 11,53749 | 1,143938 | 4,247992 | 13,48634 | 4,530245 | 4,928506 | 2,489011 | 0,354206 | 5,519266 | 12,87995 | 1,418889 | 3,621997 | 6,423862 | 1,895357 | 2,990535 | 4,611607 | 2,579604 | 3,506261 | 3,25276 | 1,550655 | 3,361019 | 4,208894 | 2,161084 |
| 02020 Two-component system | Signal transduction | 2,942107 | 3,579627 | 0,283616 | 2,812332 | 7,820745 | 2,402445 | 3,287254 | 0,920857 | 0,100631 | 3,453552 | 5,895062 | 0,887955 | 2,248441 | 2,682065 | 1,449711 | 2,310393 | 2,712883 | 1,974784 | 2,177413 | 1,523545 | 0,946077 | 2,124723 | 2,315198 | 1,843046 |
| 02024 Quorum sensing | Cellular community - prokaryotes | 10,20343 | 29,76691 | 2,547782 | 7,536057 | 29,33166 | 9,140951 | 9,104496 | 5,992121 | 0,780884 | 10,21495 | 31,34721 | 3,176293 | 7,03334 | 15,87538 | 4,895279 | 5,316894 | 10,06322 | 8,205816 | 6,001049 | 6,885338 | 3,737353 | 5,794131 | 11,32814 | 8,118764 |
| 02025 Biofilm formation - Pseudomonas aeruginosa | Cellular community - prokaryotes | 3,309405 | 4,169293 | 0,17995 | 3,158969 | 7,629213 | 1,619471 | 3,544682 | 1,152517 | 0,072623 | 4,033694 | 7,429485 | 1,094914 | 1,429367 | 1,576975 | 0,654249 | 1,796849 | 0,901575 | 0,530075 | 0,912602 | 0,760463 | 0,289718 | 0,893365 | 0,623889 | 0,365316 |
| 02026 Biofilm formation - Escherichia coli | Cellular community - prokaryotes | 4,9374 | 5,243294 | 0,336481 | 4,744722 | 10,81595 | 2,748197 | 5,580436 | 1,438004 | 0,140974 | 6,034632 | 8,525501 | 1,331186 | 2,221479 | 2,645269 | 0,831922 | 2,238945 | 2,295772 | 0,839492 | 2,379613 | 1,607069 | 0,718357 | 2,363133 | 1,52747 | 0,636367 |
| 02030 Bacterial chemotaxis | Cell motility | 3,928046 | 1,464823 | 0,054032 | 3,91783 | 11,31818 | 2,265105 | 4,910133 | 0,823183 | 0,049211 | 4,60097 | 7,731175 | 1,521931 | 1,647715 | 1,399696 | 1,172632 | 2,305659 | 1,531318 | 1,094854 | 1,770764 | 1,036331 | 0,403317 | 1,555612 | 0,866429 | 0,60156 |
| 02040 Flagellar assembly | Cell motility | 3,115322 | 2,415703 | 0,141026 | 2,909476 | 8,734076 | 1,610697 | 3,461574 | 0,993389 | 0,06839 | 3,500123 | 7,068156 | 1,148209 | 1,71842 | 2,110493 | 1,602781 | 2,090327 | 1,929466 | 1,250579 | 2,041317 | 1,356838 | 0,776932 | 1,830266 | 1,540137 | 1,021284 |
| 02060 Phosphotransferase system (PTS) | Membrane transport | 4,582898 | 20,48772 | 0,418015 | 4,157159 | 15,65759 | 4,665746 | 4,831715 | 2,111965 | 0,259742 | 5,196293 | 12,70783 | 1,21512 | 1,982553 | 12,19424 | 1,285743 | 1,570898 | 4,370542 | 1,570106 | 2,494169 | 2,708514 | 0,945716 | 2,22884 | 3,80277 | 0,928013 |
| 03008 Ribosome biogenesis in eukaryotes | Translation | 4,769833 | 5,93577 | 0,129845 | 5,143953 | 10,27846 | 2,541833 | 5,83398 | 1,954214 | 0,236494 | 5,966857 | 7,042285 | 1,250788 | 7,266515 | 5,285231 | 2,490389 | 6,574656 | 6,416995 | 1,697385 | 7,649064 | 4,772133 | 2,688556 | 7,472971 | 2,950143 | 1,581842 |
| 03010 Ribosome | Translation | 49,54492 | 139,3645 | 39,26713 | 42,88078 | 120,8229 | 75,99668 | 51,22981 | 45,00454 | 31,63614 | 47,13637 | 114,0597 | 36,99494 | 81,49415 | 121,11 | 82,39119 | 71,26815 | 100,4342 | 99,36461 | 82,76872 | 73,36291 | 73,02707 | 80,42933 | 96,45226 | 87,15598 |
| 03013 Nucleocytoplasmic transport | Translation | 0 | 0 | 0,006283 | 0 | 0 | 0 | 0 | 0,02834 | 0,010423 | 0 | 0 | 0 | 0 | 0 | 0 | 0,047498 | 0 | 0 | 0,015615 | 0 | 0 | 0,019593 | 0 | 0 |
| 03015 mRNA surveillance pathway | Translation | 0 | 0 | 0 | 0 | 0,086599 | 0 | 0 | 0 | 0 | 0 | 0,023853 | 0,017131 | NA | NA | NA | NA | NA | NA | NA | NA | NA | NA | NA | NA |
| 03018 RNA degradation | Folding, sorting and degradation | 9,511381 | 17,62623 | 1,93775 | 8,447914 | 26,49036 | 11,08006 | 11,20652 | 4,29096 | 0,684999 | 10,31605 | 18,89798 | 2,253154 | 18,50319 | 19,64072 | 18,06955 | 16,31723 | 20,70491 | 21,433 | 18,11099 | 14,03075 | 14,18825 | 18,93227 | 19,75213 | 18,56352 |
| 03020 RNA polymerase | Transcription | 25,27672 | 105,8619 | 8,517035 | 16,27299 | 89,13366 | 27,61462 | 23,60263 | 19,77836 | 2,406825 | 21,18898 | 91,16503 | 6,44954 | 39,59474 | 74,49626 | 38,11099 | 30,59434 | 58,07521 | 52,57888 | 37,46788 | 36,35486 | 32,51437 | 36,1151 | 51,07763 | 45,16493 |
| 03030 DNA replication | Replication and repair | 10,21849 | 41,00459 | 3,521245 | 7,460451 | 36,82749 | 12,21107 | 9,132758 | 7,921544 | 0,981372 | 9,63437 | 37,20845 | 3,053382 | 17,18462 | 32,94103 | 14,60607 | 13,43732 | 27,45671 | 24,91282 | 14,94073 | 14,77123 | 10,26302 | 15,20389 | 28,00366 | 26,04036 |
| 03050 Proteasome | Folding, sorting and degradation | 10,87727 | 75,70816 | 6,625062 | 3,218471 | 46,67861 | 5,018283 | 5,064651 | 15,31911 | 2,083817 | 6,084258 | 67,26846 | 3,393855 | 11,34439 | 31,44553 | 4,047621 | 3,869231 | 16,11186 | 2,200394 | 7,874577 | 14,64429 | 5,351381 | 6,491108 | 12,22728 | 2,757575 |
| 03060 Protein export | Folding, sorting and degradation | 13,89636 | 51,21833 | 3,540605 | 8,963729 | 43,18047 | 10,04027 | 12,35364 | 9,628463 | 1,309337 | 12,2793 | 46,44533 | 4,234987 | 16,30436 | 39,0424 | 15,5973 | 12,65719 | 28,06001 | 26,68634 | 13,532 | 16,43751 | 11,83547 | 13,30569 | 29,21176 | 26,9201 |
| 03070 Bacterial secretion system | Membrane transport | 8,556533 | 24,95211 | 1,479652 | 6,274224 | 23,92474 | 4,740024 | 7,685137 | 4,752974 | 0,70635 | 8,377973 | 25,2633 | 2,705444 | 8,773394 | 19,65828 | 8,038388 | 7,445909 | 13,58653 | 13,26942 | 7,202038 | 8,30375 | 5,772465 | 7,246846 | 14,29528 | 13,34954 |
| 03410 Base excision repair | Replication and repair | 4,442244 | 10,86151 | 1,249528 | 4,216975 | 18,85799 | 10,21098 | 5,332887 | 2,185799 | 0,317141 | 5,208705 | 10,56028 | 1,361464 | 7,661361 | 15,54656 | 8,880549 | 6,905342 | 17,40804 | 18,20139 | 8,123662 | 6,027196 | 5,070078 | 7,853104 | 16,56065 | 17,84575 |
| 03420 Nucleotide excision repair | Replication and repair | 9,661936 | 36,38036 | 4,603057 | 7,013098 | 40,7091 | 19,55599 | 9,260623 | 8,122434 | 1,097757 | 8,922487 | 33,30471 | 2,301621 | 20,0405 | 29,11549 | 15,85882 | 15,12477 | 30,75276 | 23,49489 | 19,10327 | 16,31604 | 11,55168 | 18,52361 | 25,64245 | 20,79885 |
| 03430 Mismatch repair | Replication and repair | 8,318568 | 25,7828 | 2,450904 | 6,967744 | 27,50155 | 11,8706 | 8,304011 | 5,061104 | 0,633036 | 8,758787 | 25,32564 | 2,523973 | 13,65433 | 25,52706 | 12,27279 | 11,25807 | 22,41006 | 22,00822 | 12,13495 | 11,37405 | 8,226642 | 12,61015 | 24,09958 | 23,30045 |
| 03440 Homologous recombination | Replication and repair | 11,91612 | 45,87263 | 3,788576 | 8,843616 | 39,44201 | 13,58934 | 10,94293 | 8,642401 | 1,117685 | 11,45214 | 40,74071 | 3,504281 | 20,32526 | 39,01652 | 19,31329 | 15,65654 | 32,70519 | 31,73893 | 17,95497 | 17,94602 | 13,73333 | 18,24403 | 33,10187 | 30,9191 |
| 03450 Non-homologous end-joining | Replication and repair | 0 | 0 | 0 | 0 | 0,08805 | 0 | 0 | 0 | 0 | 0,014591 | 0 | 0 | 0,450559 | 0,384516 | 0,261321 | 0,887584 | 0,373055 | 0,242364 | 0,09605 | 0,200267 | 0,05954 | 0,045699 | 0,261705 | 0,245693 |
| 04010 MAPK signaling pathway | Signal transduction | 0 | 0 | 0 | 0 | 0 | 0 | 0 | 0,178971 | 0 | 0 | 0 | 0 | NA | NA | NA | NA | NA | NA | NA | NA | NA | NA | NA | NA |
| 04011 MAPK signaling pathway - yeast | Signal transduction | 3,420072 | 1,341441 | 0,062994 | 3,390118 | 5,927066 | 1,572256 | 3,520445 | 0,633765 | 0,018901 | 4,242627 | 5,759003 | 1,121031 | 12,34364 | 3,482634 | 3,078787 | 12,15161 | 7,457409 | 4,583278 | 9,332572 | 3,815253 | 3,577104 | 10,029 | 5,946229 | 2,452529 |
| 04013 MAPK signaling pathway - fly | Signal transduction | 5,451952 | 0,514002 | 0,00577 | 5,746548 | 8,555719 | 0,973394 | 8,202927 | 0,24738 | 0,077489 | 7,526516 | 2,372231 | 0,629258 | 21,32134 | 5,075931 | 9,458235 | 17,81887 | 16,22414 | 9,226287 | 22,54365 | 7,01894 | 7,359545 | 22,15419 | 7,747433 | 6,115079 |
| 04014 Ras signaling pathway | Signal transduction | 0 | 0 | 0 | 0 | 0 | 0 | 0 | 0,178971 | 0 | 0 | 0 | 0 | NA | NA | NA | NA | NA | NA | NA | NA | NA | NA | NA | NA |
| 04015 Rap1 signaling pathway | Signal transduction | 0 | 0,008733 | 0,022915 | 0 | 0,070152 | 0,009328 | 0 | 0,092727 | 0,007751 | 0 | 0,031439 | 0,008326 | 0 | 0 | 0 | 0 | 0 | 0 | 0 | 0 | 0 | 0 | 0,017539 | 0 |
| 04016 MAPK signaling pathway - plant | Signal transduction | 7,962931 | 2,961705 | 0,853578 | 8,191745 | 16,89537 | 8,511486 | 9,123681 | 1,219299 | 0,070783 | 10,20159 | 12,90274 | 2,62315 | 11,95839 | 3,471526 | 5,190775 | 10,85234 | 8,487102 | 5,787559 | 11,83662 | 3,716111 | 4,262768 | 11,76242 | 5,822452 | 3,655306 |
| 04022 cGMP-PKG signaling pathway | Signal transduction | 0 | 0 | 0 | 0 | 0,086599 | 0 | 0 | 0 | 0 | 0 | 0,023853 | 0,017131 | NA | NA | NA | NA | NA | NA | NA | NA | NA | NA | NA | NA |
| 04024 cAMP signaling pathway | Signal transduction | 0 | 0 | 0 | 0 | 0,086599 | 0 | 0 | 0 | 0 | 0 | 0,023853 | 0,017131 | NA | NA | NA | NA | NA | NA | NA | NA | NA | NA | NA | NA |
| 04066 HIF-1 signaling pathway | Signal transduction | 10,01156 | 32,03457 | 3,358273 | 8,081929 | 40,89396 | 16,20922 | 10,92064 | 7,246812 | 1,188452 | 9,963569 | 31,56547 | 2,858932 | 17,51161 | 20,26872 | 13,45053 | 14,27489 | 20,78629 | 12,4871 | 16,87187 | 13,20906 | 10,77695 | 17,11049 | 15,43949 | 9,850284 |
| 04068 FoxO signaling pathway | Signal transduction | 7,054707 | 1,684108 | 0,062994 | 7,22115 | 11,61661 | 2,217751 | 8,989063 | 0,739028 | 0,07056 | 9,266878 | 7,340491 | 1,540536 | 16,83249 | 4,279282 | 6,268511 | 14,98524 | 11,84077 | 6,904783 | 15,93811 | 5,417097 | 5,468325 | 16,0916 | 6,846831 | 4,283804 |
| 04070 Phosphatidylinositol signaling system | Signal transduction | 6,813709 | 7,616747 | 1,979956 | 6,688096 | 21,12694 | 14,20987 | 6,560157 | 2,014207 | 0,359724 | 8,16757 | 15,33006 | 2,079378 | 6,135501 | 5,129886 | 4,239947 | 5,167975 | 8,792035 | 3,989401 | 5,325695 | 3,533768 | 3,140107 | 5,281812 | 5,811427 | 1,555715 |
| 04072 Phospholipase D signaling pathway | Signal transduction | 2,336357 | 1,279783 | 0,047323 | 2,442633 | 5,678485 | 1,716734 | 2,464873 | 0,421902 | 0,098888 | 2,919654 | 5,019729 | 0,944223 | 1,157617 | 1,875736 | 0,423188 | 1,550392 | 1,380788 | 0,713908 | 0,56142 | 0,665189 | 0 | 0,604848 | 4,058513 | 0,572645 |
| 04080 Neuroactive ligand-receptor interaction | Signaling molecules and interaction | 0 | 0,106669 | 2,005607 | 0,036192 | 3,337273 | 17,05813 | 0 | 0 | 0 | 0,018338 | 0,39088 | 0,147474 | 4,8094 | 1,196834 | 5,444519 | 5,850263 | 1,299151 | 3,569285 | 3,896213 | 2,460487 | 2,564174 | 4,648543 | 1,312292 | 2,012916 |
| 04110 Cell cycle | Cell growth and death | 0 | 0 | 0 | 0 | 0,021268 | 0 | 0 | 0 | 0 | 0 | 0 | 0 | NA | NA | NA | NA | NA | NA | NA | NA | NA | NA | NA | NA |
| 04112 Cell cycle - Caulobacter | Cell growth and death | 6,879603 | 19,41944 | 1,981305 | 6,054722 | 22,2463 | 9,328768 | 8,232941 | 4,53095 | 0,545939 | 7,367412 | 18,73563 | 1,954914 | 16,32579 | 19,52418 | 14,38471 | 14,17664 | 20,73976 | 19,18144 | 16,23911 | 12,48919 | 11,17665 | 16,23324 | 18,58577 | 17,03653 |
| 04113 Meiosis - yeast | Cell growth and death | 0 | 0 | 0 | 0,010664 | 0,323704 | 0 | 0,01281 | 0 | 0 | 0 | 0,035883 | 0,008566 | 0,083617 | 0 | 0 | 0,158314 | 0 | 0 | 0 | 0,158474 | 0,158477 | 0,022322 | 0 | 0 |
| 04114 Oocyte meiosis | Cell growth and death | 0 | 0 | 0 | 0 | 0,053934 | 0 | 0 | 0 | 0 | 0 | 0,011927 | 0,008566 | NA | NA | NA | NA | NA | NA | NA | NA | NA | NA | NA | NA |
| 04115 p53 signaling pathway | Cell growth and death | 0 | 0 | 0 | 0,169604 | 1,787664 | 0,04634 | 0,098529 | 0 | 0 | 0 | 0,147563 | 0,01296 | 1,365696 | 0,419769 | 0,52814 | 1,659387 | 0,300607 | 0,358539 | 0,248961 | 0,02554 | 0 | 0,060994 | 0 | 0,240097 |
| 04120 Ubiquitin mediated proteolysis | Folding, sorting and degradation | 0 | 0,002075 | 0,004467 | 0 | 0,054741 | 0,031091 | 0 | 0,006014 | 0 | 0 | 0,022002 | 0,006732 | 0 | 0 | 0 | 0 | 0 | 0,00135 | 0 | 0 | 0 | 0 | 0,003287 | 0 |
| 04122 Sulfur relay system | Folding, sorting and degradation | 6,092887 | 2,123792 | 0,430746 | 6,316507 | 13,79059 | 5,88219 | 7,222793 | 1,176484 | 0,107223 | 7,655653 | 9,378566 | 1,933268 | 4,757622 | 2,201357 | 2,559034 | 5,397333 | 4,081413 | 2,422697 | 5,213751 | 2,397906 | 1,879807 | 5,27118 | 3,648151 | 1,928266 |
| 04137 Mitophagy - animal | Transport and catabolism | 0 | 0,002593 | 0,002699 | 0 | 0,063044 | 0,036288 | 0 | 0,007517 | 0 | 0 | 0,027503 | 0,008415 | 0 | 0 | 0 | 0 | 0 | 0,00135 | 0 | 0 | 0 | 0 | 0,003287 | 0 |
| 04138 Autophagy - yeast | Transport and catabolism | 11,69579 | 81,88665 | 9,004885 | 3,275374 | 48,91413 | 20,12614 | 5,012279 | 15,79934 | 1,954821 | 5,947567 | 68,6985 | 3,471393 | 12,59219 | 34,42228 | 5,662896 | 4,640508 | 17,92757 | 4,099867 | 8,786424 | 15,83802 | 5,90418 | 7,815691 | 13,59084 | 2,90625 |
| 04141 Protein processing in endoplasmic reticulum | Folding, sorting and degradation | 0,333164 | 0,104302 | 0,861033 | 0,532287 | 3,957261 | 8,240567 | 1,154431 | 0 | 0 | 0,600271 | 1,676398 | 0,350244 | 10,95124 | 3,436534 | 7,212426 | 9,734912 | 7,931596 | 6,595733 | 10,83468 | 4,794336 | 4,618424 | 11,09796 | 6,413729 | 4,195962 |
| 04142 Lysosome | Transport and catabolism | 1,311543 | 6,395602 | 1,238289 | 1,177892 | 5,066192 | 6,212638 | 1,494068 | 1,419603 | 0,158917 | 1,096528 | 6,145749 | 0,332512 | 16,19673 | 29,81589 | 18,77396 | 13,52562 | 33,09975 | 41,40541 | 15,28976 | 9,87657 | 9,652966 | 15,70613 | 38,18813 | 46,10586 |
| 04144 Endocytosis | Transport and catabolism | 0 | 0,002042 | 0 | 0 | 0,009059 | 0 | 0 | 0,089485 | 0 | 0,000652 | 0,000814 | 0 | NA | NA | NA | NA | NA | NA | NA | NA | NA | NA | NA | NA |
| 04145 Phagosome | Transport and catabolism | 0 | 0,004367 | 0,011458 | 0 | 0,104374 | 0,004664 | 0 | 0,022373 | 0,008422 | 0 | 0,021907 | 0,004163 | 0 | 0 | 0 | 0 | 0 | 0 | 0 | 0 | 0 | 0 | 0,017539 | 0 |
| 04146 Peroxisome | Transport and catabolism | 5,009891 | 11,70121 | 1,034563 | 3,91483 | 13,95465 | 1,817808 | 4,992417 | 2,513281 | 0,299934 | 5,215024 | 14,02501 | 1,288209 | 8,742181 | 10,4814 | 5,016786 | 6,787887 | 10,59562 | 8,873754 | 7,495849 | 5,502449 | 3,615563 | 7,766471 | 10,93006 | 10,27028 |
| 04151 PI3K-Akt signaling pathway | Signal transduction | 0,301978 | 0 | 0 | 0,505047 | 1,480733 | 0,212487 | 0,859474 | 0 | 0 | 0,593507 | 0,72109 | 0,17436 | 16,30803 | 4,632542 | 7,92053 | 13,20643 | 12,26936 | 6,660523 | 15,75743 | 6,037086 | 5,524932 | 15,85358 | 9,100036 | 4,520211 |
| 04152 AMPK signaling pathway | Signal transduction | 3,555195 | 0,810714 | 0,046472 | 3,66453 | 6,918734 | 1,144575 | 4,767072 | 0,717053 | 0,320185 | 4,51841 | 2,477992 | 0,639187 | 16,08356 | 7,558959 | 13,54259 | 14,17454 | 13,00861 | 13,28642 | 14,00979 | 8,387257 | 10,9638 | 16,24414 | 11,22929 | 10,72823 |
| 04210 Apoptosis | Cell growth and death | 0,020668 | 0,005822 | 0,015277 | 0,248696 | 1,522655 | 0,247424 | 0,494671 | 0,002161 | 0,005167 | 0 | 0,659709 | 0,13101 | 1,908335 | 0,718982 | 1,094388 | 1,898103 | 0,420968 | 0,317793 | 1,864468 | 0,678539 | 1,06049 | 1,382391 | 0,48488 | 0,475234 |
| 04214 Apoptosis - fly | Cell growth and death | 3,008285 | 0,989248 | 1,618653 | 3,11313 | 8,941573 | 13,42585 | 3,305116 | 0,343724 | 0,043437 | 3,312263 | 3,831177 | 0,773766 | 3,371598 | 1,276583 | 3,253452 | 4,34776 | 1,300694 | 2,542939 | 2,780927 | 1,716292 | 1,811295 | 2,877423 | 1,932235 | 1,232563 |
| 04215 Apoptosis - multiple species | Cell growth and death | 0 | 0 | 0 | 0,169604 | 1,787664 | 0,04634 | 0,098529 | 0 | 0 | 0 | 0,147563 | 0,01296 | 1,365696 | 0,419769 | 0,52814 | 1,659387 | 0,300607 | 0,358539 | 0,248961 | 0,02554 | 0 | 0,060994 | 0 | 0,240097 |
| 04216 Ferroptosis | Cell growth and death | 13,53068 | 31,55205 | 2,838365 | 10,67094 | 35,14204 | 5,132586 | 11,62447 | 7,159267 | 0,880733 | 13,91027 | 40,67028 | 3,987495 | 22,2631 | 40,70718 | 18,77184 | 16,19685 | 40,36923 | 43,51639 | 18,62011 | 17,54788 | 11,5788 | 20,02765 | 45,63421 | 53,05814 |
| 04217 Necroptosis | Cell growth and death | 9,753039 | 38,63009 | 2,836896 | 7,426252 | 28,41261 | 7,147702 | 9,491827 | 7,353997 | 0,883967 | 9,485555 | 34,15907 | 2,77248 | 18,14562 | 26,35469 | 12,39209 | 14,22474 | 20,08319 | 13,64218 | 17,71443 | 14,95434 | 10,47728 | 16,62581 | 17,07521 | 9,394626 |
| 04218 Cellular senescence | Cell growth and death | 0 | 0 | 0 | 0 | 0,086599 | 0 | 0 | 0 | 0 | 0 | 0,023853 | 0,017131 | NA | NA | NA | NA | NA | NA | NA | NA | NA | NA | NA | NA |
| 04370 VEGF signaling pathway | Signal transduction | 0 | 0 | 0 | 0 | 0 | 0 | 0 | 0,178971 | 0 | 0 | 0 | 0 | NA | NA | NA | NA | NA | NA | NA | NA | NA | NA | NA | NA |
| 04390 Hippo signaling pathway | Signal transduction | 0 | 0,005822 | 0,015277 | 0 | 0,082724 | 0,006219 | 0 | 0,002161 | 0,005167 | 0 | 0,02891 | 0,011261 | 0 | 0 | 0 | 0 | 0 | 0 | 0 | 0 | 0 | 0 | 0,017539 | 0 |
| 04391 Hippo signaling pathway - fly | Signal transduction | 0 | 0,008733 | 0,022915 | 0 | 0,080786 | 0,009328 | 0 | 0,003241 | 0,007751 | 0 | 0,031439 | 0,008326 | 0 | 0 | 0 | 0 | 0 | 0 | 0 | 0 | 0 | 0 | 0,017539 | 0 |
| 04510 Focal adhesion | Cellular community - eukaryotes | 0 | 0,005822 | 0,015277 | 0 | 0,075634 | 0,006219 | 0 | 0,061818 | 0,005167 | 0 | 0,02891 | 0,011261 | 0 | 0 | 0 | 0 | 0 | 0 | 0 | 0 | 0 | 0 | 0,017539 | 0 |
| 04520 Adherens junction | Cellular community - eukaryotes | 0 | 0,008733 | 0,022915 | 0 | 0,070152 | 0,009328 | 0 | 0,092727 | 0,007751 | 0 | 0,031439 | 0,008326 | 0 | 0 | 0 | 0 | 0 | 0 | 0 | 0 | 0 | 0 | 0,017539 | 0 |
| 04530 Tight junction | Cellular community - eukaryotes | 0 | 0,008733 | 0,022915 | 0 | 0,070152 | 0,009328 | 0 | 0,092727 | 0,007751 | 0 | 0,031439 | 0,008326 | 0 | 0 | 0 | 0 | 0 | 0 | 0 | 0 | 0 | 0 | 0,017539 | 0 |
| 04540 Gap junction | Cellular community - eukaryotes | 0 | 0 | 0 | 0 | 0,069837 | 0 | 0 | 0 | 0 | 0 | 0 | 0 | NA | NA | NA | NA | NA | NA | NA | NA | NA | NA | NA | NA |
| 04810 Regulation of actin cytoskeleton | Cell motility | 0 | 0,005822 | 0,015277 | 0 | 0,075634 | 0,006219 | 0 | 0,061818 | 0,005167 | 0 | 0,02891 | 0,011261 | 0 | 0 | 0 | 0 | 0 | 0 | 0 | 0 | 0 | 0 | 0,017539 | 0 |
| 05111 Biofilm formation - Vibrio cholerae | Cellular community - prokaryotes | 4,464133 | 5,024609 | 0,282007 | 4,258774 | 10,37996 | 2,649964 | 5,117522 | 1,087328 | 0,116369 | 5,324238 | 7,377803 | 1,197842 | 3,25628 | 3,751879 | 1,90159 | 3,711822 | 2,951122 | 2,107408 | 3,098405 | 2,124724 | 1,363527 | 3,203327 | 2,300394 | 1,729398 |
| 04071 Sphingolipid signaling pathway | Signal transduction | NA | NA | NA | NA | NA | NA | NA | NA | NA | NA | NA | NA | 0,22569 | 0,090888 | 0,133243 | 0,361548 | 0,08633 | 0,085905 | 0 | 0 | 0 | 0 | 0 | 0,025101 |
