# Supplementary material for: Sugar lowering in fermented apple-pear juice orchestrates a promising metabolic answer in the gut microbiome and intestinal integrity
Source: Curr Res Food Sci. 2024 Sep 5;9:100833. doi: 10.1016/j.crfs.2024.100833 (PMC11406026; doi:10.1016/j.crfs.2024.100833)
Supplement: Multimedia component 6 [file mmc6.docx]

**Table S4.** Sensory properties of raw apple-pear juice (FJC), apple-pear juice fermented with *Apilactobacillus kunkeei* BEE4 for 96 h (FJL), apple-pear juice fermented with *Saccharomyces cerevisiae* KFAY2 for 72 h (FJY), and apple-pear juice fermented sequentially by *A. kunkeei* BEE4 for 72 h and then followed by *S. cerevisiae* KFAY2for 48 h (FJSeq), respectively. Fermented apple-pear juices were incubated at 30 °C. Column data with different superscript letters (a-c) differ significantly (P < 0.05).

| Samples | Appearance | Aroma | Flavor | Acidity | Sweetness | Astringency | Overall rating |
| --- | --- | --- | --- | --- | --- | --- | --- |
| FJC | 8.7 ± 1.0 | 7.2 ± 1.2 | 7.2 ± 1.1^ab^ | 2.2 ± 1.0^c^ | 8.9 ± 0.7^a^ | 1.3 ± 0.5 | 7.8 ± 1.0 |
| FLJ | 8.5 ± 1.3 | 8.1 ± 1.3 | 8.0 ± 1.0^a^ | 5.9 ± 1.0^a^ | 6.1 ± 0.9^b^ | 1.7 ± 1.0 | 7.6 ± 1.2 |
| FJY | 7.6 ± 0.9 | 7.0 ± 0.9 | 6.3 ± 1.1^b^ | 2.0 ± 1.0^c^ | 2.7 ± 1.1^c^ | 1.4 ± 0.5 | 6.8 ± 1.4 |
| FJSeq | 7.9 ± 1.3 | 6.9 ± 1.0 | 6.3 ± 0.8^b^ | 4.3 ± 0.9^b^ | 4.0 ± 1.3^c^ | 1.5 ± 0.7 | 6.9 ± 1.0 |
